# Genome-wide association study identifies four pan-ancestry loci for suicidal ideation in the Million Veteran Program

**DOI:** 10.1371/journal.pgen.1010623

**Published:** 2023-03-20

**Authors:** Allison E. Ashley-Koch, Nathan A. Kimbrel, Xue J. Qin, Jennifer H. Lindquist, Melanie E. Garrett, Michelle F. Dennis, Lauren P. Hair, Jennifer E. Huffman, Daniel A. Jacobson, Ravi K. Madduri, Hilary Coon, Anna R. Docherty, Jooeun Kang, Niamh Mullins, Douglas M. Ruderfer, the VA Million Veteran Program (MVP), the MVP Suicide Exemplar Workgroup, the International Suicide Genetics Consortium, Philip D. Harvey, Benjamin H. McMahon, David W. Oslin, Elizabeth R. Hauser, Michael A. Hauser, Jean C. Beckham

**Affiliations:** 1 Duke Molecular Physiology Institute, Durham, North Carolina, United States of America; 2 Department of Medicine, Duke University Health System, Durham, North Carolina, United States of America; 3 Durham Veterans Affairs (VA) Health Care System, Durham, North Carolina, United States of America; 4 VA Mid-Atlantic Mental Illness Research, Education and Clinical Center, Durham, North Carolina, United States of America; 5 VA Health Services Research and Development Center of Innovation to Accelerate Discovery and Practice Transformation, Durham, North Carolina, United States of America; 6 Department of Psychiatry and Behavioral Sciences, Duke University School of Medicine, Durham, North Carolina, United States of America; 7 Massachusetts Veterans Epidemiology Research and Information Center (MAVERIC), VA Boston Healthcare System, Boston, Massachusetts, United States of America; 8 Biosciences, Oak Ridge National Laboratory, Oak Ridge, TN, United States of America; 9 Bredesen Center for Interdisciplinary Research and Graduate Education, University of Tennessee Knoxville, Knoxville, Tennessee, United States of America; 10 Department of Psychology, NeuroNet Research Center, University of Tennessee Knoxville, Knoxville, Tennessee, United States of America; 11 Consortium for Advanced Science and Engineering, The University of Chicago, Chicago, Illinois, United States of America; 12 Data Science and Learning Division, Argonne National Laboratory, Lemont, Illinois, United States of America; 13 Department of Psychiatry, Huntsman Mental Health Institute, University of Utah School of Medicine, Salt Lake City, Utah, United States of America; 14 Biomedical Informatics, University of Utah School of Medicine, Salt Lake City, Utah, United States of America; 15 Department of Psychiatry, Virginia Commonwealth University, Richmond, Virginia, United States of America; 16 Division of Genetic Medicine, Department of Medicine, Vanderbilt Genetics Institute, Vanderbilt University Medical Center, Nashville, Tennessee, United States of America; 17 Department of Genetics and Genomic Sciences, Icahn School of Medicine at Mount Sinai, New York, New York, United States of America; 18 Department of Psychiatry, Icahn School of Medicine at Mount Sinai, New York, New York, United States of America; 19 Department of Biomedical Informatics, Vanderbilt University Medical Center, Nashville, Tennessee, United States of America; 20 Department of Psychiatry and Behavioral Sciences, Vanderbilt University Medical Center, Nashville, Tennessee, United States of America; 21 Research Service Bruce W. Carter VA Medical Center, Miami, Florida, United States of America; 22 Department of Psychiatry and Behavioral Sciences, University of Miami Miller School of Medicine, Miami, Florida, United States of America; 23 Theoretical Biology and Biophysics, Los Alamos National Laboratory, Los Alamos, New Mexico, United States of America; 24 VISN 4 Mental Illness Research, Education, and Clinical Center, Center of Excellence, Corporal Michael J. Crescenz VA Medical Center, Philadelphia, Pennsylvania, United States of America; 25 Department of Psychiatry, Perelman School of Medicine, University of Pennsylvania, Pennsylvania, United States of America; 26 Department of Biostatistics and Bioinformatics, Duke University School of Medicine, Durham, North Carolina, United States of America; National Cancer Institute, UNITED STATES

## Abstract

Suicidal ideation (SI) often precedes and predicts suicide attempt and death, is the most common suicidal phenotype and is over-represented in veterans. The genetic architecture of SI in the absence of suicide attempt (SA) is unknown, yet believed to have distinct and overlapping risk with other suicidal behaviors. We performed the first GWAS of SI without SA in the Million Veteran Program (MVP), identifying 99,814 SI cases from electronic health records without a history of SA or suicide death (SD) and 512,567 controls without SI, SA or SD. GWAS was performed separately in the four largest ancestry groups, controlling for sex, age and genetic substructure. Ancestry-specific results were combined via meta-analysis to identify pan-ancestry loci. Four genome-wide significant (GWS) loci were identified in the pan-ancestry meta-analysis with loci on chromosomes 6 and 9 associated with suicide attempt in an independent sample. Pan-ancestry gene-based analysis identified GWS associations with *DRD2*, *DCC*, *FBXL19*, *BCL7C*, *CTF1*, *ANNK1*, and *EXD3*. Gene-set analysis implicated synaptic and startle response pathways (q’s<0.05). European ancestry (EA) analysis identified GWS loci on chromosomes 6 and 9, as well as GWS gene associations in *EXD3*, *DRD2*, and *DCC*. No other ancestry-specific GWS results were identified, underscoring the need to increase representation of diverse individuals. The genetic correlation of SI and SA within MVP was high (r_G_ = 0.87; p = 1.09e-50), as well as with post-traumatic stress disorder (PTSD; r_G_ = 0.78; p = 1.98e-95) and major depressive disorder (MDD; r_G_ = 0.78; p = 8.33e-83). Conditional analysis on PTSD and MDD attenuated most pan-ancestry and EA GWS signals for SI without SA to nominal significance, with the exception of *EXD3* which remained GWS. Our novel findings support a polygenic and complex architecture for SI without SA which is largely shared with SA and overlaps with psychiatric conditions frequently comorbid with suicidal behaviors.

## Author summary

Suicidal ideation often precedes and predicts suicide attempt and death, is the most common suicidal phenotype and is over-represented in veterans. The underlying genetic risk of suicidal ideation in the absence of suicide attempt is unknown, yet believed to have distinct and overlapping risk with other suicidal behaviors. To find these potential genetic risk variants, we examined blood samples from over 600,000 veterans from the Million Veteran Program. Four regions of the genome were significantly associated with risk of suicidal ideation in the absence of suicide attempt across four major ancestral groups (European, African, Hispanic and Asian). Two of those regions on chromosomes 6 and 9, were also significantly associated with suicidal ideation in European ancestry-only veterans, as well as with suicide attempt in an independent sample of mostly civilian individuals. No other genetic regions were identified in other ancestry-only analyses, underscoring the need to increase representation of individuals with diverse ancestry. Suicidal ideation without suicide attempt was genetically correlated with other psychiatric disorders, including suicide attempt, post-traumatic stress disorder and major depressive disorder. Our novel findings suggest that multiple genes contribute to genetic risk for suicidal ideation and that the genetic risk is largely shared with suicide attempt and other psychiatric conditions that frequently co-occur with suicidal ideation.

## Introduction

Rates of suicide have steadily increased in the U.S. during the past 20 years [1,2], particularly among veterans [1]. As a result, suicide is now the second leading cause of death among Iraq/Afghanistan-era veterans who served after September 11, 2001, accounting for 22.3% of all deaths within this cohort (https://stopsoldiersuicide.org/vet-stats). Rates of suicidal ideation (SI) also appear to be increasing among U.S. adults [3]. The percentage U.S. adults who reported SI in the past year increased from 3.7 percent (or 8.3 million people) in 2008 to 4.8 percent (or 12.0 million people) in 2019 [3]. Rates of SI are even higher among veterans. For example, Smith and colleagues reported that 8.7–9.9% of veterans from a nationally-representative sample endorsed SI in the past two weeks [4]. Importantly, SI often precedes and predicts both fatal and non-fatal suicide attempts [5]. In fact, SI was the third strongest prospective predictor of death by suicide identified by the largest meta-analysis of longitudinal risk factors for suicide to date [5].

Given the higher prevalence of SI relative to suicide deaths and suicide attempts [3] and the robust prospective association between SI and death by suicide [5], identification of biomarkers for SI in the absence of suicide attempt has potential to contribute to ongoing suicide prevention efforts. Moreover, prior research demonstrates that SI is likely to have both shared and distinct genetic risk with suicide attempts and suicide deaths [6,7]. A family study found that suicide attempts were elevated among first-degree relatives of adolescents who had died by suicide relative to controls, even after adjusting for the presence of other psychiatric disorders, whereas the increased rate of SI observed among relatives of probands appeared to be due to higher rates of psychiatric disorders [7]. In contrast, a more recent study that utilized registry data from Sweden reported that parental psychiatric and substance use disorders explained up to 40% of the genetic transmission effects observed for suicide attempts [8]. Some twin studies suggest that heritability could actually be greater for SI and suicide attempts than for death by suicide. For example, Fu et al. reported unadjusted heritability estimates for suicidal ideation and attempt of 47% and 30%, respectively, among male veterans [6]. Moreover, after adjustment, estimates were still 36% and 17%, respectively, providing clear evidence that genetic effects on SI are likely to be independent of psychopathology. The different heritability estimates for SI and suicide attempts suggest that SI in the absence of attempt is not simply a less severe form of suicide attempt, and clinically, not all individuals with SI will necessarily go on to make a suicide attempt.

Unfortunately, despite its potential clinical significance, little is known about the molecular genetic architecture of SI without attempt. While several genome-wide association studies (GWAS) have included SI as part of the definition of a broader suicide phenotype, [9–11] or have studied treatment emergent suicidal ideation within the context of clinical trials, [12] we are unaware of any GWAS that has identified genome-wide significant (GWS) associations with SI excluding suicide attempts and deaths. One recent study in the UK Biobank identified GWS loci on chromosomes 9, 11, and 13 using a combined “suicidality” phenotype that included SI, deliberate self-harm, and previous attempts [10]. A related study in the UK Biobank focused on “self-harm ideation”, which included suicide attempt and non-suicidal self-harm, identified a single GWS locus on chromosome 5 [13]. Notably, the UK Biobank studies were the largest to include SI as part of the definition of the outcome to date, which is consistent with the observation that larger sample sizes are crucial for discovering novel, replicable risk loci through GWAS in relation to complex phenotypes [14].

The objective of the present research was to conduct the largest GWAS of SI without attempt to date within the Million Veterans Program (MVP) cohort (99,814 SI cases; 512,567 controls) in order to identify pan-ancestry and ancestry-specific loci associated with risk for SI, specifically, among U.S. military veterans, a population at markedly increased risk for SI and death by suicide.

## Materials and methods

### Study participants and ethics statement

Since 2011, the MVP study has enrolled over 825,000 veteran participants from across the U.S. in order to understand the interplay between genes, lifestyle and military exposures on health. Participation in the study included written informed consent, completion of questionnaires, donating a blood sample for genetic analysis and linking the biorepository data to the VA electronic health records (EHR) system. The MVP EHR-biorepository is one of the largest and most diverse for the examination of genetic contributions to human traits, particularly ones that are important to veterans’ health. This study was approved by the Department of Veteran’s Affairs Central Institutional Review Board (Central VA IRB number 18–11).

### Suicidal Ideation phenotype and comorbid conditions

Veterans were classified as cases if there was any indication of SI in their electronic health records (EHR) and they did not have a history of suicide attempts (*N* = 21,899) nor indeterminate behavior/death in SPAN (n = 226) or NDI (n = 14,431). This resulted in 99,814 cases of SI without SA. Four sources from the U.S. Department of Veterans Affairs (VA) EHR were utilized to create the SI phenotype, including: (a) International Classification of Diseases (ICD9 and ICD10) codes for intentional self-harm; (b) suicide behavior reports from the VA’s Suicide Prevention Applications Network (SPAN) database [15]; (c) mental health survey responses from the VA’s Mental Health Assistant database indicating thoughts of either active or passive SI; and (d) VA specific extract from the National Death Index (NDI) (see **S1 Text, S1–S3 Tables** for additional details). 21.1% of SI cases were identified by more than one source, 12.9% were identified by diagnostic codes only, 0.3% were identified by SPAN records only, and 65.8% were identified by mental health surveys only. Individuals without genotype information and/or without ancestry designation (see Genetic substructure section below) were excluded from analysis, as well. Veteran participants were classified as controls (N = 512,567) if they had no documented lifetime history of SI or suicidality based on qualifying ICD codes, suicide behavior reports, or mental health survey responses.

Given the frequent comorbidity of SI with other psychiatric conditions, we also determined the presence of bipolar disorder (BPD), major depressive disorder (MDD), schizophrenia (SCZ) and post-traumatic stress disorder (PTSD) among SI cases and controls. Qualifying ICD codes for these four conditions were obtained from the MVP Phenomics library [16]. To ensure the accuracy of the comorbid condition, we required at least one inpatient stay due to the comorbid diagnosis or at least two outpatient visits due to the comorbid diagnosis in the same calendar year. Alternatively, the participant could have at least two EHR records of having submitted a request to the VA for reimbursement of visits to a community provider for treatment of the comorbid diagnosis in the same calendar year.

### MVP genotyping and imputation

The genotyping methods and quality control (QC) procedures for the MVP genotype data have been described elsewhere, [17] but included genotyping on a custom Axiom 1.0 array, as well as exclusion of samples of questionable identity and with genotyping call rates below 98.5%. The original QC analysis of the data set by the MVP Release 4 data team also evaluated the data set for related individuals. In the case that a third-degree or closer relative was identified, one individual of that pair was removed from the data set. Imputation of the genotyping data was performed with the global reference panel from 1000Genomes [18]. Subsequent to all these procedures performed by the MVP Release 4 data team, additional QC in the present analysis included excluding markers with a minor allele frequency (MAF) < 0.01 in the entire MVP data set to focus on common genetic variation, as well as excluding markers with an imputation INFO score <0.6 in order to utilize markers with a high degree of confidence. After all these exclusions, 13,615,936 markers were available for the present meta-analysis.

### Statistical methods

#### Genetic substructure

We performed principal component analysis (PCA) using PLINK2 [19] and non-imputed genotypes within each of the four largest, mutually-exclusive ancestral groups assigned through a prior MVP study focused on harmonizing genetic ancestry and self-identified race/ancestry (HARE) [[15,20]]: European-Ancestry, African-Ancestry, Hispanic-Ancestry, and Asian-Ancestry. The HARE ancestry assignment used both genetic markers and self-report to assign individuals to the four ancestral groups. To further control for population substructure within ancestral group, we used 20 principal components (PC’s) for the European-Ancestry subset (EA; lambda_GC_ = 1.20 after PC adjustment), 6 for the African-Ancestry subset (AA; lambda_GC_ = 1.04), 8 for the Hispanic-Ancestry subset (HA; lambda_GC_ = 1.06), and 6 for the Asian-Ancestry subset (AS; lambda_GC_ = 0.97).

#### Genetic association and meta-analysis

Ancestry-specific GWAS was performed using PLINK2 [19], controlling for age, sex and genetic PC’s. Meta-analysis was performed with the R package metafor [21]. GWAS results were visualized using Manhattan plots, along with LocusZoom plots [22] for specific associated genomic regions. The QE-test of heterogeneity of effect sizes for the genome-wide significant hits (GWS) were performed with the R package metafor [21]. In addition, forest plots of the GWS hits were also constructed.

#### Replication

GWS associations identified in the MVP were tested for replication in the International Suicide Genetics Consortium (ISGC), using results from their recent GWAS of suicide attempts, in a large, international sample of primarily civilian individuals (N = 549,743 total subjects, N = 29,782 cases) and EA descent (90%) [11]. Replication was performed for MVP meta-analysis as well as for European-American ancestry-specific GWS loci in the ISGC. In most cases, the GWS SNP was available in the ISGC. In the cases where the GWS SNP was not available in ISGC, we attempted to identify a proxy SNP with r^2^>0.5 or D’ = 1 in the EA population using LDproxy [23,24]. In total, we performed look-ups in ISGC for six GWS markers, giving a Bonferroni correction for replication of 0.008 (0.05/6).

The Mid-Atlantic MIRECC cohort [25] was used as a target sample to test polygenic risk scores (PRS) generated from the MVP SI GWAS results. Briefly, the MIRECC cohort includes *N* = 2,423 U.S. military veterans, many of whom experienced combat and have histories of PTSD, depression, and suicide attempts [25–27]. SI cases were defined in the MIRECC as having SI only (i.e., no history of suicide attempt). This resulted in *N* = 331 EA cases and *N* = 847 EA controls, and *N* = 334 AA cases and *N* = 911 AA controls. Effect sizes from the MVP GWAS for the EA and AA subsets were used to generate PRS to test for association with SI in the comparable MIRECC ancestral groups using the program PRSice [28] with default parameters for clumping using the linkage disequilibrium (LD) patterns from the MIRECC subset (EA or AA) under investigation. The clumping process eliminates redundant SNPs in high LD [29]. PRSs were computed from all SNPs with p-values from the discovery (MVP) GWAS that fell below a particular threshold. Since the p-value threshold which will maximize the predictive power in the target data set (MIRECC) is not known *a priori*, PRSs were calculated for 1,001 thresholds in the MVP ranging from p = 0.0001 to 1, in increments of 0.001. Empirical p-values were computed for the predictions due to the smaller sample size of the target data set.

#### SNP heritability and genetic correlation

Linkage disequilibrium score regression (LDSC) [30,31] was used to estimate the SNP heritability of SI among the EA subset of MVP, as well as that heritability conditioned on insomnia (https://nealelab.github.io/UKBB_ldsc/h2_summary_1200.html), BPD [32], MDD [33], SCZ [34] and PTSD [35]. To calculate heritability on the liability scale, we used a population prevalence of 0.09 [4] and a sample prevalence of 0.14. LDSC was also used to estimate genetic correlation between SI in the EA subset of MVP with suicide attempts both in the previously published GWAS studies for the MVP [9] and the ISGC [11], as well as with conditions frequently comorbid with SI including insomnia, BPD, MDD, SCZ and PTSD. Finally, the SNP heritability for the AA and HA subsets of MVP were computed using GCTA [36].

#### Conditional analyses based on common comorbid conditions

Analysis of the SI GWAS results conditioned on insomnia, BPD, MDD, SCZ and PTSD GWAS results was performed with mtCOJO as implemented in GCTA [37]. Since the GWAS summary statistics for the exposure trait (comorbid condition) and the outcome trait (SI) needed to be LD matched and from the same ancestry, we used the SI GWAS results from the EA subset for the conditional analyses. The exposure traits were derived from previously published GWAS studies for insomnia (https://nealelab.github.io/UKBB_ldsc/h2_summary_1200.html), BPD [32], MDD [33], SCZ [34] and PTSD [35]. To perform the analysis, independent SNPs were selected as instruments, as defined by LD r^2^<0.05 using the 1000 Genomes Project Phase 3 EUR reference panel [38]. To obtain at least 10 independent SNPs across all five comorbid conditions, we set the genome-wide significance threshold to p<1.0x10^-5^. This provided 105 index SNPs for insomnia, 65 index SNPs for BPD, 34 index SNPs for MDD, 365 index SNPs for SCZ, and 11 index SNPs for PTSD.

#### GWAS functional mapping and annotation

To perform annotation and enrichment tests of the pan-ancestry and EA GWAS results for SI, we used FUMA [39] with the EA LD structure. FUMA aids in prioritizing the putative causal genes and SNPs within GWAS loci by integrating data from multiple data repositories [39]. MAGMA [40] gene-based and gene-set analysis was evaluated through FUMA. Gene-based tests were performed for 19,216 genes (Bonferroni-corrected p-value threshold = 2.602E-06). 10678 gene sets (curated gene sets: 4761, GO terms: 5917) pre-defined from MsigDB v6.2 were evaluated for association with SI (Bonferroni-corrected p-value threshold = 4.7E-06). Tissue-set enrichment analyses were also performed using MAGMA implemented in FUMA, to test for enrichment of association signal in genes expressed in 30 general tissue types from GTEx v7 (Bonferroni-corrected p-value threshold = 1.7E-03). Genes within the identified risk loci were also tested for enrichment of previously reported GWAS catalog associations.

## Results

### Demographics

As shown in **Table 1**, the present data set included 99,814 SI cases and 512,567 controls making it the largest analysis of SI to date. As we have previously reported for suicide attempts in MVP [9], the frequency of SI differed significantly by ancestral group (19.0% in Asian-Ancestry, 14.1% in European-Ancestry, 22.5% in Hispanic-Ancestry, 21.6% in African-Ancestry; p < 2.2e-16), as well. However, whereas suicide attempts were more frequent in individuals of African-Ancestry, SI was most frequent among individuals of Hispanic-Ancestry. As previously reported, [41,42] SI was also more frequent among women (23.2% females vs. 15.6% males; p < 2.2e-16) and younger individuals (55.8 yr cases vs. 62.96 yr controls; p < 2.2e-16). These differences in frequency of SI by ancestry, gender and age in the MVP do not necessarily reflect population-based estimates, however, because participants in the MVP were self-selected rather than epidemiologically-selected for study participation.

Comorbidity with other psychiatric conditions was common (**Table 1**). In particular, more than half the SI cases were comorbid for MDD or PTSD. Although the comorbidity of BPD and SCZ with SI was not as common as MDD or PTSD, these conditions were also more frequent among SI cases than controls. SI case status was generally associated with a medium to large effect size (as measured by standardized mean difference; **Table 1**) on the presence of BPD, MDD, PTSD and SCZ.

**Table 1:** *Sample characteristics*.

	Controls	Ideation Only Cases	Standardized Mean Difference^1^
**Total (%)**	512,567 (83.7)	99,814 (16.3)	
**Age** (mean (SD))	62.96 (13.84)	55.80 (13.67)	0.520
**Age Group** (%)			0.555
18–29	12,285 (2.4)	4,511 (4.5)	
30–39	28,245 (5.5)	10,986 (11.0)	
40–49	40,337 (7.9)	13,591 (13.6)	
50–59	84,571 (16.5)	24,186 (24.2)	
60 and over	347,129 (67.7)	46,540 (46.7)	
**Male (**%)	471,103 (91.9)	87,321 (87.5)	0.146
**Psychiatric Comorbidity (%)**			
Bipolar disorder	16371 (3.2%)	14708 (14.7%)	0.413
Major depressive disorder	119212 (23.3%)	73610 (73.7%)	1.171
Post-traumatic stress disorder	91337 (17.8%)	58196 (58.3%)	0.917
Schizophrenia	8295 (1.6%)	6340 (6.4%)	0.244
**HARE Ancestry Group** (%)			0.247
European-American	376,826 (73.5)	62,023 (62.1)	
African-American	90,835 (17.7)	25,097 (25.1)	
Asian-American	6,528 (1.3)	1,529 (1.5)	
Hispanic-American	38,378 (7.5)	11,165 (11.2)	
**Self-Reported Race**^**2**^ **(%)**			
White (only)	385,636 (75.2)	65,613 (65.7)	0.209
Black (only)	85,753 (16.7)	23,695 (23.7)	0.175
Asian (only)	5,429 (1.1)	1,251 (1.3)	0.018
**Self-Reported Ethnicity** (%)			0.118
Hispanic or Latino/a	37,547 (7.3)	10,652 (10.7)	
Not Hispanic or Latino/a	469,319 (91.6)	88,238 (88.4)	
**Military Service**^**3**^ **(%)**			
September 2001 or later (%)	57,456 (11.2)	17,844 (17.9)	0.193
August 1990 to August 2001 (includes Persian Gulf War) (%)	114,166 (22.3)	32,907 (33.0)	0.245
May 1975 to July 1990 (%)	116,582 (22.7)	25,602 (25.6)	0.073
Prior Feb1975 (includes Vietnam, Korea, World War II) (%)	333,270 (65.0)	45,962 (46.0)	0.392

*Notes*: ^1^Standardized mean differences of 0.2, 0.5, and 0.8 can be interpreted as small, medium, and large effects sizes, respectively. ^2^ Self-reported race utilized a “mark all that apply format,” we report here results for participants who endorsed only one race category to facilitate comparison with the ancestral groups that were utilized, which were mutually exclusive. ^3^Military service utilized a “mark all that apply format.” Thus, these categories are not mutually exclusive.

### Pan-ancestry GWAS results and annotation

**Fig 1:**
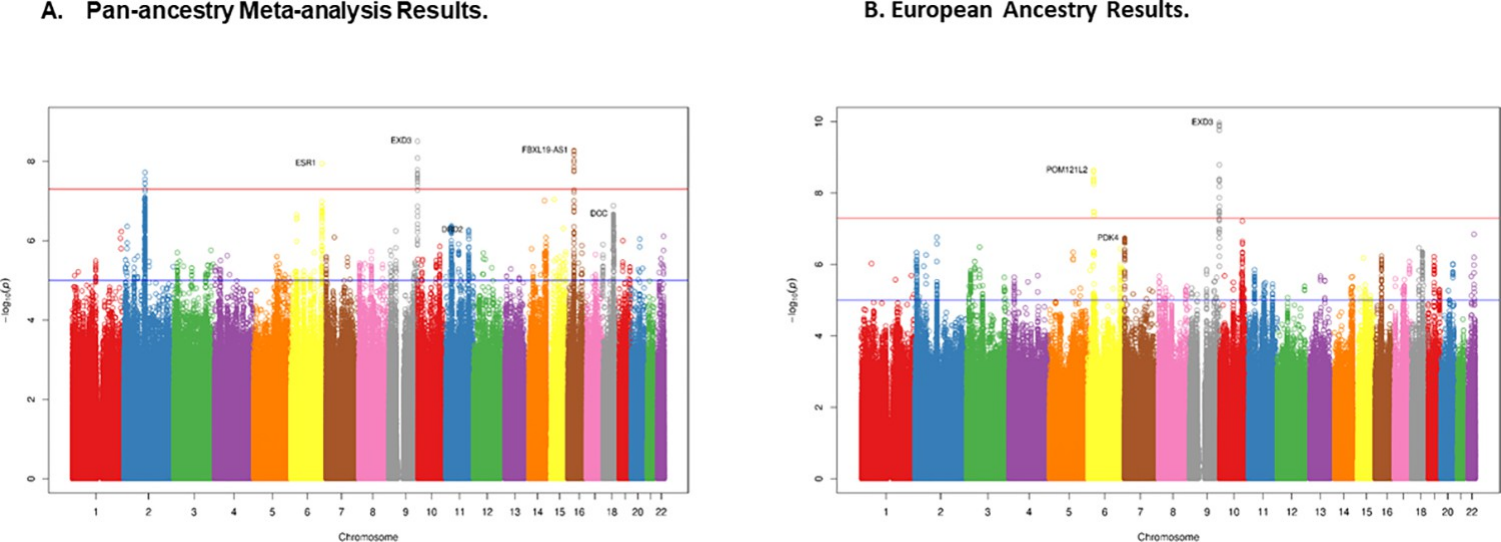
Single Marker GWAS Results for Suicidal Ideation in the Million Veterans Program. A. Pan-ancestry Meta-analysis Results. B. European Ancestry Results.

Meta-analysis across the four major ancestral groups in MVP identified a total of four genome-wide significant (GWS) loci for SI (**Table 2**; **Fig 1A**). These are the first reported GWS loci derived from a GWAS specifically of SI. Formal tests of heterogeneity were performed for the four GWS loci, none of which were statistically significant, suggesting that these loci are relevant for all four MVP ancestries examined. Ancestry-specific allele frequencies for these markers can be found in **S4 Table**. Forest plots of these loci can be found in **S1 Fig**. We attempted to replicate these GWS loci in the ISGC GWAS of suicide attempts [11]. The most significant GWS SNP on chromosomes 6 and 9 replicated in the ISGC (**Table 2**), even after Bonferroni correction for multiple testing (q = 0.05/6 = 0.008). For the chromosomes 2 and 16 loci, we did not observe association with suicide attempt. For the chromosome 2 locus, a proxy SNP in high LD (r^2^ = 0.9) with the MVP GWS SNP was used for the replication attempt. Locus Zoom plots of the associated regions can be found in **S2 Fig**.

**Table 2:** Genome-wide Significant (p<5E-8) Loci for Single Marker GWAS of Suicidal Ideation.

**SNP**	**Chromosome: Position^+^**	**Alleles eff/alt**	**p-value**	**Effect Size**	**Annotation (gene)**	Replication in ISGC* (**proxy SNP)	**Test for heterogeneity** **(p-value)**
**Meta-Analysis Pan-ancestry Results (99,814 cases, 512,567 controls)**							
rs77641763	9:140265782	T/C	4.43E-10	0.059 (0.010)	*EXD3*	2.55E-03	0.37
rs7185007	16:30927509	C/T	1.24E-08	0.035 (0.006)	*FBXL19-AS1*	2.59E-01	0.23
rs142785607	2:104267493	T/G	1.50E-08	0.031 (0.005)	*AC018880*.*2*	1.32E-01 (rs67716713)	0.91
rs6557168	6:152201201	C/T	1.67E-08	0.032 (0.006)	*ESR1*	7.73E-03	0.76
**SNP**	**Chromosome: Position** ^**+**^	**Alleles eff/alt**	**p-value**	**Odds Ratio (SE)**	**Annotation (gene)**	**Replication in ISGC**	**Test for heterogeneity** **(p-value)**
**European Ancestry Results (62,023 cases, 376,826 controls)**							
rs73581580	9:140251458	A/G	1.08E-10	1.07 (0.01)	*EXD3*	1.23E-03	3.1E-03
rs13211166	6:27265940	A/G	2.34E-09	0.94 (0.01)	*POM121L2*	4.84E-07	3.5E-05

+Using GRCh37(hg19) genome build.

* Bonferroni correction for six marker look-ups in ISGC resulted in q = 0.008 (p = 0.05/6).

**If exact SNP was not available in the ISGC data set, we utilized LDproxy to identify a proxy SNP in high LD (r^2^ > 0.5).

**Fig 2:**
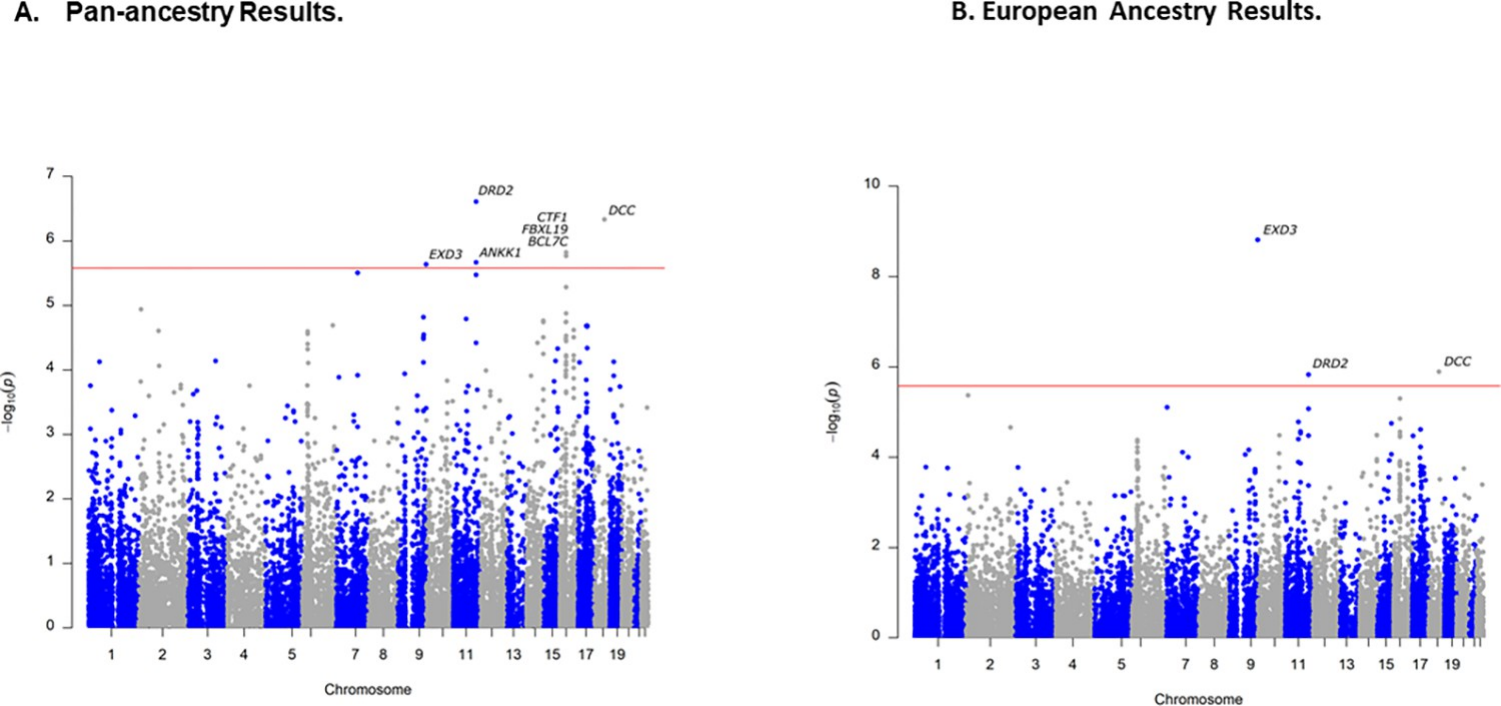
Gene-based Manhattan Plots. A. Pan-ancestry Results. B. European Ancestry Results.

**Fig 3:**
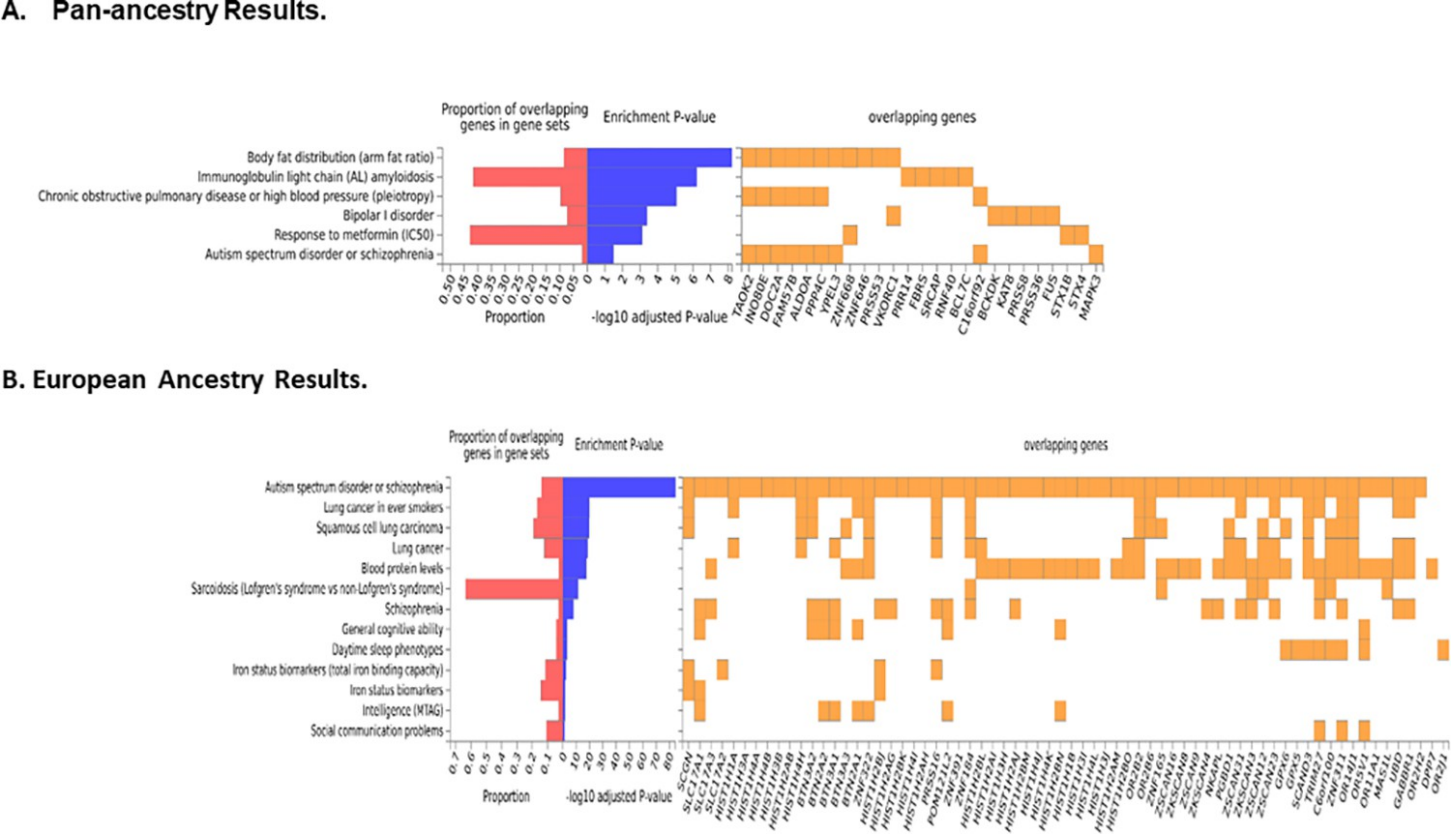
Enrichment of GWAS Catalog Terms. A. Pan-ancestry Results. B. European Ancestry Results.

Using FUMA, [39] we identified seven GWS genes associated with SI in the gene-based tests, including *EXD3* on chromosome 9, *DRD2* and *ANNK1* on chromosome 11, *DCC* on chromosome 18, as well as *FBXL19*, *BCL7C*, and *CTF1* all on chromosome 16 (**Fig 2A**). The genes on chromosome 16 and *EXD3* on chromosome 9 were represented by single marker GWS associations (**Table 2**). However, the other GWS genes were not represented by single marker GWS loci and reflected the accumulation of nominally significant associations across those genes. Gene-set analysis using the entire GWAS results revealed a significant enrichment of GO terms for synapse (p = 1.28E-06; q = 0.02) and startle response (p = 2.03E-06; q = 0.03), as well as an enrichment for expression in brain (p = 0.002), but no other general tissue types. Finally, the GWS genes were also enriched for several terms in the GWAS catalog, most notably bipolar disorder, as well as autism spectrum disorder or schizophrenia, but also several other terms such as body fat distribution and response to the anti-diabetic medication metformin (**Fig 3A**).

### Ancestry-specific GWAS results and annotation

The EA subset is the largest ancestral group present in MVP and thus, the subset with the greatest amount of statistical power. For the EA subset, we identified two GWS loci associated with SI on chromosomes 6 and 9 (**Table 2; Fig 1B**). The GWS loci on chromosomes 6 and 9 replicated in the ISGC, even after correction for multiple testing (**Table 2**). Of note, the peak marker on chromosome 9 was different than the peak marker in the pan-ancestry analysis, however, these two markers are in complete LD among Europeans (CEU r^2^ = 1.0). Ancestry-specific allele frequencies for these markers can be found in **S4 Table**. Forest plots of the locus (**S1A and S1E Fig**), visualize that the reason for this discrepancy is due to the AA group. In fact, the LD of these two markers in Africans is much lower (YRI r^2^ = 0.04). In the follow-up analyses with FUMA, [39] the gene-based analysis identified three GWS gene associations: *EXD3* (chromosome 9), *DRD2* (chromosome 11), and *DCC* (chromosome 18) (**Fig 2B**). Of these, *EXD3* was represented in the single marker GWS loci for the pan-ancestry and EA subset GWAS (**Fig 1A and 1B**). All three genes were also GWS in the pan-ancestry gene-based analysis (**Fig 2A**), suggesting that the EA subset of the MVP was driving those gene-based associations. Regional plots of the two EA GWS loci are provided in **S2F and S2G Fig**). None of the gene-set analyses were significant after multiple testing correction and there was no tissue enrichment observed in the EA subset. However, several GWAS catalog terms were enriched, including autism spectrum disorder or schizophrenia, as well as cognitive ability and intelligence, among others (**Fig 3B**).

No GWS loci were identified in the other ancestral groups (AA, AS and HA) and thus, no additional annotation analyses with FUMA [39] were performed. The Manhattan plots for these additional ancestral groups are provided in **S3A and S3C Fig**.

### PRS with the MIRECC data set

The MVP EA subset GWAS results were utilized to predict SI in the MIRECC EA subset, but none of the MVP p-value thresholds were statistically significant in the MIRECC EA subset. The most significant threshold was identified with MVP EA p’s < 0.66 (PRS empirical p = 0.57; **S4A Fig**). This threshold explained only 0.2% of the variability in SI on the liability scale. Similarly, although no GWS loci were identified in the AA subset of MVP, we utilized summary statistics from that ancestry-specific analysis to predict SA in the AA subset of the MIRECC data set. None of those MVP p-values were statistically significant in the MIRECC AA subset. However, the most predictive threshold was with MV AA p’s < 0.83 trended towards significance (PRS empirical p = 0.09; **S4**B **Fig**). The most predictive threshold explained less than 0.6% of the variability in SI on the liability scale.

### Genetic heritability of SI and genetic correlation between SI and suicide attempts

Within the EA subset of the MVP, we estimated the SNP heritability of SI on the liability scale to be 0.046 (se 0.003; **Table 3**). This was slightly higher than our previous estimate of suicide attempts in the MVP (0.013; se 0.002) [9]. We also note that although our λ_GC_ for EA = 1.20, the intercept was 1.04, suggesting that the inflation that we were observing was not due to population stratification, but due to polygenicity [31]. Using GCTA, we estimated the SNP heritabilities for the next two largest ancestral subgroups (AA and HA). The estimates of SI SNP heritability for the AA subset (0.036; se 0.006; **Table 3**) was slightly lower than the heritability estimate of SI in the EA subset. The estimate of SI SNP heritability for the HA subset was larger (0.108), but had a wider standard error (0.010) due to the smaller size of the HA subset. Given that the sample size of the AS subset was smaller than the HA, we did not estimate the SNP heritability of SI in that subset. These data, however, generally suggest that there are likely similar genetic architectures for SI across ancestral groups. The low SNP heritability estimates (<0.1) suggest that additive models of common SNP contributions to risk are likely insufficient to explain the genetic architecture of SI.

Also using the EA subset of the MVP, we tested for the genetic correlation between the outcome of SI with SA in the MVP, as well as the independent, mostly EA ISGC data set. Both these correlations were high (r_G_>0.75; **Table 3**). Nonetheless, the correlation of SI in MVP with SA in ISGC was slightly lower than the genetic correlation of SA in the MVP with SA in the ISGC (r_G_ = 0.86; p = 1.30e-21) which we previously observed [9], suggesting that there is at least some unique genetic risk to SI that is independent of suicide attempt.

**Table 3:** Heritability of Suicidal Ideation in the Million Veterans Program and Genetic Correlation with Frequent Comorbid Conditions.

**Heritability of Suicidal Ideation**		
**Ancestral Group**	**Conditioned on Comorbid Condition**	**Liability-based Heritability Estimate (SE)**
EA		0.046 (0.003)
	BPD	0.045 (0.003)
	Insomnia	0.046 (0.003)
	MDD	0.030 (0.003)
	PTSD	0.023 (0.003)
	SCZ	0.042 (0.003)
AA		0.036 (0.006)
HA		0.108 (0.01)
**Genetic Correlation of Suicidal Ideation in EA subset with Other Phenotypes**		
**Correlated Phenotype**	**Genetic Correlation (SE)**	**p-value**
Suicide Attempts in MVP	0.87 (0.06)	1.09E-50
Suicide Attempts in ISGC	0.77 (0.05)	2.15E-53
BPD	0.25 (0.04)	1.17E-08
Insomnia	0.08 (0.02)	0.0009
MDD	0.78 (0.04)	8.33E-83
PTSD	0.78 (0.04)	1.98E-95
SCZ	0.32 (0.04)	1.71E-19

### Effects of comorbidity on SI genetic architecture

We also tested the genetic correlation of SI in the MVP subset with common comorbid conditions (BPD, MDD, PTSD, SCZ and insomnia; **Table 3**). The genetic correlations of SI with both MDD and PTSD were high (r_G_>0.75; **Table 3**), but lower for the other three conditions (r_G_<0.35; **Table 3**). Consistent with this observation, conditioning the heritability of SI on MDD and PTSD had the largest effect on the heritability estimate (**Table 3**). The heritability of SI was reduced by a third when conditioning on MDD and by a half when conditioning on PTSD. Although these were sizable reductions, the conditioned heritability estimates suggest that SI is nonetheless still heritable above and beyond comorbidity with these conditions. The heritability of SI conditioned on BPD, SCZ and insomnia had a negligible effect on the SI heritability estimates.

The GWS signals were also evaluated conditioned on these comorbid conditions (**Table 4**). To perform this analysis, all unconditioned p-values were obtained from only the EA subset of MVP, even for the pan-ancestry GWS loci, so that there was a consistent LD map for SI and the comorbid GWAS. Remarkably, the chromosome 9 locus (*EXD3*) remained GWS regardless of the comorbid conditioning. The significance of the chromosome 16 locus (*FBXL19-AS1*) was minimally affected by the conditioning, reducing by an order of magnitude when conditioned by most of the comorbid conditions, but was least affected by conditioning on BPD. The significance of the remaining loci on chromosomes 2 (*AC018880*.*2*) and 6 (*ESR1*, *POM121L2*) were reduced most strongly by conditioning on PTSD and MDD, consistent with the strong genetic correlation that we had observed between those conditions and SI.

**Table 4:** Genome-wide Significant Loci for Suicidal Ideation Conditioned on Comorbid Conditions.

		SI Unconditioned*	SI Conditioned on Comorbid Condition				
SNP	Annotation	p-value	Insomnia (p-value)	PTSD (p-value)	SCZ (p-value)	BPD (p-value)	MDD (p-value)
rs73581580	*EXD3*	1.08E-10	2.47E-10	**	1.75E-10	8.55E-11	3.27E-09
rs77641763	*EXD3*	1.75E-10	3.99E-10	**	3.44E-10	1.29E-10	2.31E-09
rs13211166	*POM121L2*	2.34E-09	1.94E-09	9.94E-06	6.65E-07	1.04E-08	1.94E-07
rs6557168	*ESR1*	3.64E-07	3.84E-07	2.80E-05	5.59E-07	6.24E-07	1.41E-05
rs7185007	*FBXL19-AS1*	8.21E-07	1.27E-06	6.42E-06	1.05E-06	5.76E-07	5.98E-06
rs142785607	*AC018880*.*2*	4.09E-06	3.01E-06	8.92E-06	3.55E-06	**	0.0002

* Unconditioned p-values were those obtained with EA subset only. Thus, the GWS SNPs for the pan-ancestry meta-analysis signals were pulled from the EA subset results for the conditioning analyses. This was because the comorbid condition GWAS results used in GCTA were all from EA analyses.

**Marker not present in the comorbid condition GWAS results.

## Discussion

We have conducted the first GWAS specific to suicidal ideation and identified several GWS loci and genes. Due to the richness of the VA EHR, we were able to distinguish between SI and SA, as well as identify controls with no evidence of suicidal phenotypes. This is a unique aspect of the MVP data set that has not previously been accomplished with other data sets. Within the spectrum of suicidal phenotypes, SI is the most common and often precedes and predicts fatal and non-fatal suicide attempts [5]. As a result, understanding the biologic underpinnings of SI, including the genetic basis, could be important for developing efficacious treatments and interventions for suicide.

Among the four GWS loci that we identified in the pan-ancestry analysis, two replicated in the independent ISGC data set [11], including *EXD3* on chromosome 9 and *ESR1* on chromosome 6. Neither of these loci were significantly heterogeneous across the four MVP ancestry subgroups. *EXD3* is a 3’ to 5’ exonuclease involved in nucleic acid binding, but while it has been associated with several behavioral phenotypes, its functional role in these conditions remains unclear. Importantly, a GWAS of PTSD in the MVP also detected an association with *EXD3* [43] and our lead GWS SNP (rs77641763) in *EXD3* has been previously associated with chronotype (“morningness”) [44] and insomnia [45]. As reported here, there is strong genetic correlation between SI and PTSD, but also between insomnia and suicide attempts [9,46], and we previously found that circadian rhythm pathways were one of the top pathways emerging from the MVP suicide attempts GWAS [9]. But despite these connections to PTSD and insomnia, *EXD3* was the only SI locus that remained GWS when conditioned on comorbid conditions (**Table 4**), albeit the SNP was not present in the PTSD GWAS [35]. Of note, the pan-ancestry lead SNP in *EXD3* is in high LD (r^2^ = 0.81) with a missense variant (rs79140116) in the gene, suggesting that the true functional variant may actually be rs79140116. Although in the EA subset, we observed a different lead SNP (rs73581580), this association replicated in the ISGC, as well (**Table 2**), and was also in high LD (r^2^ = 0.85) with rs79140116. Importantly, the SI locus that we observed is also distinct from the locus on chromosome 9 that was previously observed in a GWAS of suicidality in the UK Biobank [10].

The second replicated GWS locus in the pan-ancestry analysis was on chromosome 6 at the Estrogen Receptor 1 (*ESR1*) locus. The same SNP (rs6557168) that was most significantly associated with SI in the present study, was also associated with anxiety in the MVP [47] which is interesting since symptoms of anxiety have also been strongly associated with suicidality [46,48,49]. Also of note, *ESR1* has been identified as a driver causal gene in an integrated genomic analysis of individuals with depression and/or PTSD [50], two conditions frequently comorbid with SI (**Table 1**) and highly genetically correlated with SI (**Table 3**). Conditioning the *ESR1* locus on both of these comorbid conditions did slightly attenuate the signal (**Table 4**).

Among the remaining GWS loci in the pan-ancestry analysis, the chromosome 2 GWS locus is in the region of a pseudogene, *AC018880*.*2* and there were several GWS SNPs at this locus, although this locus was not associated with suicide attempts in the ISGC. Thus, additional investigation will be required to determine if this is a locus that may be specific to SI and if so, to identify the relevant gene or variant. Finally, the chromosome 16 GWS locus was also not associated with suicide attempt in the ISGC data set but contained multiple GWS SNPs in the MVP data set. This is a gene-rich region and we also identified GWS gene associations at this locus with *FBXL19*, *BCL7C* and *CTF1*, all three genes being reasonable candidates with putative roles in neuronal development, neuronal degeneration and/or brain cancer [51–56].

In the EA subset, the two GWS loci we identified replicated in the ISGC, including *EXD3* as discussed above, and *POM121L2*. *POM121L2* encodes a constituent of the nuclear pore, but otherwise, little is known about its function. Variants in *POM121L2* have been previously associated with risk for schizophrenia [57, 58], and we did observe an enrichment of schizophrenia GWAS loci in the EA subset (**Fig 3B**). Furthermore, conditioning this locus on SCZ attenuated the significance of this signal (**Table 4**). Notably, *POM121L2* is located in the MHC region on chromosome 6, which is quite complex and was also one of the peak regions in the ISGC GWAS of suicide attempts [11]. Also of note, the GWS locus for SA that was identified in the ISGC [11] on chromosome 7 was nominally associated in the MVP, but not GWS.

Finally, there were several gene-based GWS associations that were observed in the pan-ancestry and EA subset analyses that were not represented by single marker GWS loci. These included *DRD2* and *ANNK1* on chromosome 11, and *DCC* on chromosome 18. We previously found suggestive evidence for association of suicide attempts with *DRD2* [9]. *ANNK1* is located in the same complex region of chromosome 11, and both genes have been associated with several neuropsychiatric phenotypes [59–62]. DCC is involved in the development of the prefrontal cortex and its expression is up-regulated in brains of anti-depressant free individuals who died by suicide [63]. Further, in mouse models of defeat stress, DCC is upregulated concomitantly with downregulation of miR-218, suggesting that the co-regulation of these genes may modulate resilience and susceptibility to stress-induced psychopathology [64]. Importantly, DCC was also identified in the UK Biobank GWAS of “self-harm” ideation [13].

We acknowledge that this study has several limitations. The most significant limitation is the lack of an SI-only data set for replication. Many previous GWAS studies of suicidal phenotypes included SI as part of the phenotype under investigation, but our study is the first to restrict the GWAS to only the SI phenotype. This limitation is mitigated by the fact that we demonstrated a large degree of genetic correlation between SI in the MVP and suicide attempts in both the MVP and the ISGC (**Table 4**). Further, the fact that we were able to replicate several identified loci in the ISGC underscores the genetic overlap between the phenotypes. Nonetheless, for the loci which were not associated with suicide attempt in the ISGC, it is difficult to determine if this was due to a false positive association in the MVP data set, or if they potentially represent loci which are specific to SI rather than shared with suicide attempt. Additionally, utilizing PRS from the EA and AA subsets of MVP did not significantly predict SA in the MIRECC data set. It is unclear whether this lack of prediction was more related to overfitting the PRS to the MVP because we did not split the MVP into a training and tuning data set, or because of the significantly smaller sample size of the MIRECC data set. Collection and analysis of other data sets are needed to further explore these possibilities. Additionally, due to the size of the MVP data set, the phenotype of SI was necessarily identified from EHR. This was both an advantage and disadvantage. Our data set captured a population-based rather than clinical perspective of SI, yet also likely introduced some error in the phenotype definition. We suspect that this phenotyping approach most likely missed some cases of SI and misclassified them as controls. This would have the effect of reducing our statistical power to detect associations rather than introducing type I error. However, it is also possible that some controls were misclassified as cases. Further, due to the diversity in the MVP data set, we were able to demonstrate that at least four SI loci are consistent across ancestries. However, despite the diversity in MVP, the relative statistical power in the non-EA samples was significantly lower than that in the EA sample making it difficult to identify putative ancestry-specific loci. Efforts to examine suicidal phenotypes in diverse individuals must be increased. Similarly, the female representation in the MVP was low compared with the male representation. Given the female bias that we observed for SI, it will be important to expand female representation to identify possible sex-specific effects. Finally, we also acknowledge that there were several tests that were performed in this analysis, which could lead to false positive associations. GWAS was performed within several ancestral groups. The pan-ancestry and EA GWAS were the main analyses given the large sample sizes. The GWAS in AA, AS and HA were exploratory since those sample sizes were much smaller. But, it is crucial to conduct genetic studies in diverse ancestry groups and by presenting these data, hopefully efforts to study diverse ancestries will increase. All this aside, it is possible that some of our results may be false positives and thus, replication by other groups (beyond ISGC) is warranted. Despite these limitations, this GWAS is the first specific to SI and provides valuable insight into the genetic risk for SI.

Our findings support a polygenic architecture for risk of suicidal ideation which is largely shared with risk for suicide attempt, MDD and PTSD. The four GWS pan-ancestry loci do not display evidence for heterogeneity across ancestral groups. The SNP heritability estimates were also similar across ancestries, but the small magnitudes suggest that additive SNP heritability may not be a sufficient model for the genetic architecture of SI because previous estimates of the heritability for SI from twin studies have been in the range of 35 to 50% [6]. Our results also suggest that larger sample sizes will be needed to identify ancestry-specific loci for diverse ancestries. Identified GWS loci have been associated with other psychiatric phenotypes such as PTSD, depression, anxiety and schizophrenia which are often comorbid with suicidal phenotypes, adding confidence to our findings. In addition, the SI loci have been associated with circadian rhythm and cardiovascular phenotypes, which we had previously shown were enriched in loci for suicide attempts as well [9]. These findings shed light on the underlying biology of suicidal ideation and may aid in the development of treatments and interventions.

## Supporting information

S1 Text Defining Suicidal Ideation and Suicide Attempt Phenotypes in the MVP. This section provides more detailed information regarding how the suicide phenotypes were defined for analysis.
(DOCX)

S2 Text VA Million Veteran Program (MVP) Acknowledgements. (DOCX)

S3 Text MVP Suicide Exemplar Workgroup Acknowledgements. (DOCX)

S4 Text International Suicide Genetics Consortium (ISGC) Acknowledgement. (DOCX)

S1 Table ICD9 and ICD10 Codes Used to Phenotype Suicidal Ideation and Suicide Attempts. (DOCX)

S2 Table Suicide Prevention Applications Network (SPAN) Codes Used to Phenotype Suicide Attempts and Suicidal Ideation. (DOCX)

S3 Table Mental Health Survey Items Used to Phenotype Suicide Attempts and Suicidal Ideation. (DOCX)

S4 Table Ancestry-specific allele frequencies for the Genome-wide Significant Loci in Table 2. (DOCX)

S1 Fig Forest plots for genome-wide significant loci. **A.** Chromosome 9 locus from Pan-Ancestry Analysis**, B.** Chromosome 16 locus from Pan-Ancestry Analysis**, C.** Chromosome 2 locus from Pan-Ancestry Analysis**, D.** Chromosome 6 locus from Pan-Ancestry Analysis, **E.** Chromosome 9 locus for European-Ancestry Analysis, **F.** Chromosome 6 locus from European-Ancestry Analysis. These plots provide the point estimates and confidence intervals at each locus by ancestry and for the meta-analysis.
(TIF)

S2 Fig Regional plots of association for genome-wide significant loci. **A.** Chromosome 9 locus from Pan-Ancestry Analysis**, B.** Chromosome 16 locus from Pan-Ancestry Analysis**, C.** Chromosome 2 locus from Pan-Ancestry Analysis**, D.** Chromosome 6 locus from Pan-Ancestry Analysis, **E.** Chromosome 9 locus for European-Ancestry Analysis, **F.** Chromosome 6 locus from European-Ancestry Analysis.
(TIF)

S3 Fig Single Marker GWAS Results for Suicidal Ideation in the Million Veterans Program. **A.** African Ancestry Results, **B.** Asian Ancestry Results, **C.** Hispanic Ancestry Results.
(TIF)

S4 Fig MVP-based Suicidal Ideation Polygenic Risk Scores (PRS) Predicting Suicide Attempts+Ideation in the MIRECC cohort. **A.** European Ancestry Results, **B.** African Ancestry Results.
(TIF)
