# Supplementary material for: Genome-wide association study identifies four pan-ancestry loci for suicidal ideation in the Million Veteran Program
Source: PLoS Genet. 2023 Mar 20;19(3):e1010623. doi: 10.1371/journal.pgen.1010623 (PMC10063168; doi:10.1371/journal.pgen.1010623)
Supplement: S1 Text — This section provides more detailed information regarding how the suicide phenotypes were defined for analysis. (DOCX) [file pgen.1010623.s001.docx]

**Supplemental Methods and Results for Ashley-Koch et al**

**Defining Suicidal Ideation and Suicide Attempt Phenotypes in the MVP**

Four different electronic health record (EHR) sources were utilized to create the suicide attempt phenotype in the present study, including: (a) International Classification of Diseases (ICD9 and ICD10) codes for intentional self-harm; (b) suicide behavior reports from the VA’s Suicide Prevention Applications Network (SPAN) database; (c) mental health survey responses indicating a history of suicidal ideation (see **S1_Table, S2_Table and S3_Table** for details) (d) VA extract of the National Death Index. Veteran participants were classified as suicidal ideation cases if their EHR contained one or more: ICD-9/ICD-10 suicidal ideation codes (see **S1_Table**); SPAN reports of suicide ideation (see **S2_Table**); mental health survey responses in which participants endorsed a history of suicidal thoughts (see **S3_Table**). Veteran participants were classified as controls if they had no documented lifetime history of suicide attempts or suicidal ideation based on qualifying ICD codes, suicide behavior reports, or mental health survey responses. Note that veterans who had a history of suicidal attempts, or suspicious/indeterminate death, were excluded from the present analyses to ensure that control participants did not have a history of suicidal attempts.
