# Supplementary material for: Genome-wide association study identifies four pan-ancestry loci for suicidal ideation in the Million Veteran Program
Source: PLoS Genet. 2023 Mar 20;19(3):e1010623. doi: 10.1371/journal.pgen.1010623 (PMC10063168; doi:10.1371/journal.pgen.1010623)
Supplement: S3 Text — (DOCX) [file pgen.1010623.s003.docx]

**^22^ MVP Suicide Exemplar Workgroup Acknowledgements**

The MVP Suicide Exemplar Workgroup for this publication includes Khushbu Agarwal, Allison E. Ashley-Koch, Mihaela Aslan, Jean C. Beckham, Edmond Begoli, Tanmoy Bhattacharya, Ben Brown, Patrick S. Calhoun, Mikaela Cashman McDevitt, Kei-Hoi Cheung, Sutanay Choudhury, Ashley M. Cliff, Judith D. Cohn, Silvia Crivelli, Leticia Cuellar-Hengartner, Haedi E. Deangelis, Michelle F. Dennis, Sayera Dhaubhadel, Patrick D. Finley, Kumkum Ganguly, Michael R. Garvin, Joel E. Gelernter, Lauren P. Hair, Phillip D. Harvey, Elizabeth R. Hauser, Michael A. Hauser, Nick W. Hengartner, Daniel A. Jacobson, Piet C. Jones, David Kainer, Alan D. Kaplan, Ira R. Katz, Rachel L. Kember, Nathan A. Kimbrel, Angela C. Kirby, John C. Ko, Beauty Kolade, John Lagergren, Matthew Lane, Daniel F. Levey, Drew Levin, Jennifer H. Lindquist, Xianlian Liu, Ravi K. Madduri, Carrie Manore, Susana B. Martins, John F. McCarthy, Benjamin H. McMahon, J. Izaak Miller, Destinee Morrow, David W. Oslin, Mirko Pavicic, John P. Pestian, Saiju Pyarajan, Xue J. Qin, Nallakkandi Rajeevan, Christine M. Ramsey, Ruy Ribeiro, Jonathon Romero, Alex Rodriguez, Daniel Santel, Noah Schaefferkoetter, Yunling Shi, Murray B. Stein, Kyle A. Sullivan, Ning Sun, Suzanne R. Tamang, Alice Townsend, Jodie A. Trafton, Angelica Walker, Xiange Wang, Victoria Wangia-Anderson, Renji Yang, Shinjae Yoo, Hong-Jun Yoon, Rafael Zamora-Resendiz, and Hongyu Zhao.
