# Supplementary material for: Genome-wide association study identifies four pan-ancestry loci for suicidal ideation in the Million Veteran Program
Source: PLoS Genet. 2023 Mar 20;19(3):e1010623. doi: 10.1371/journal.pgen.1010623 (PMC10063168; doi:10.1371/journal.pgen.1010623)
Supplement: S4 Text — (DOCX) [file pgen.1010623.s004.docx]

**^23^ International Suicide Genetics Consortium (ISGC) Acknowledgements**

Niamh Mullins 1, 2, Jooeun Kang 3, Adrian I Campos 4, 5, Jonathan R I Coleman 6, 7, Alexis C Edwards 8, Hanga Galfalvy 9, 10, Daniel F Levey 11, 12, Adriana Lori 13, Andrey Shabalin 14, Anna Starnawska 15, 16, 17, 18, Mei-Hsin Su 19, Hunna J Watson 20, 21, 22, Mark Adams 23, Swapnil Awasthi 24, Michael Gandal 25, Jonathan D Hafferty 23, Akitoyo Hishimoto 26, Minsoo Kim 25, Satoshi Okazaki 27, Ikuo Otsuka 10, 27, Stephan Ripke 24, 28, 29, Erin B Ware 30, 31, Andrew W Bergen 32, 33, Wade H Berrettini 34, Martin Bohus 35, Harry Brandt 36, Xiao Chang 37, Wei J Chen 19, 38, 39, Hsi-Chung Chen 39, Steven Crawford 36, Scott Crow 40, Emily DiBlasi 14, Philibert Duriez 41, 42, Fernando Fernández-Aranda 43, Manfred M Fichter 44, 45, Steven Gallinger 46, Stephen J Glatt 47, Philip Gorwood 41, 42, Yiran Guo 37, Hakon Hakonarson 37, 48, Katherine A Halmi 49, Hai-Gwo Hwu 50, Sonia Jain 51, Stéphane Jamain 52, Susana Jiménez-Murcia 43, Craig Johnson 53, Allan S Kaplan 54, 55, 56, Walter H Kaye 57, Pamela K Keel 58, James L Kennedy 54, 55, 56, Kelly L Klump 59, Robert D Levitan 54, 55, 56, Dong Li 37, Shih-Cheng Liao 39, Klaus Lieb 60, Lisa Lilenfeld 61, Chih-Min Liu 39, Pierre J Magistretti 62, 63, Christian R Marshall 64, James E Mitchell 65, Eric T Monson 14, Richard M Myers 66, Dalila Pinto 1, 2, Abigail Powers 13, Nicolas Ramoz 42, Stefan Roepke 67, Alessandro Rotondo 68, Vsevolod Rozanov 69, 70, Stephen W Scherer 71, Christian Schmahl 35, Marcus Sokolowski 72, Michael Strober 73, 74, Laura M Thornton 22, Janet Treasure 75, 76, Ming T Tsuang 77, Maria C La Via 22, Stephanie H Witt 78, D Blake Woodside 55, 56, 79, 80, Zeynep Yilmaz 22, 81, 82, Lea Zillich 78, Rolf Adolfsson 83, Ingrid Agartz 84, 85, 86, Tracy M Air 87, Martin Alda 88, 89, Lars Alfredsson 90, 91, Ole A Andreassen 92, 93, Adebayo Anjorin 94, Vivek Appadurai 95, 96, María Soler Artigas 97, 98, 99, 100, Sandra Van der Auwera 101, M Helena Azevedo 102, Nicholas Bass 103, Claiton HD Bau 104, 105, Bernhard T Baune 106, 107, Frank Bellivier 108, 109, 110, 111, Klaus Berger 112, Joanna M Biernacka 113, Tim B Bigdeli 114, 115, Elisabeth B Binder 13, 116, Michael Boehnke 117, Marco P Boks 118, Rosa Bosch 97, 98, 119, David L Braff 120, Richard Bryant 121, Monika Budde 122, Enda M Byrne 123, 124, Wiepke Cahn 125, Miguel Casas 97, 98, 100, 119, Enrique Castelao 126, Jorge A Cervilla 127, Boris Chaumette 128, 129, 130, Sven Cichon 131, 132, 133, 134, Aiden Corvin 135, Nicholas Craddock 136, David Craig 137, Franziska Degenhardt 134, Srdjan Djurovic 138, 139, Howard J Edenberg 140, 141, Ayman H Fanous 114, 115, Jerome C Foo 142, Andreas J Forstner 131, 134, 143, Mark Frye 144, Janice M Fullerton 145, 146, Justine M Gatt 121, 145, Pablo V Gejman 147, 148, Ina Giegling 149, 150, Hans J Grabe 101, Melissa J Green 145, 151, Eugenio H Grevet 152, 153, Maria Grigoroiu-Serbanescu 154, Blanca Gutierrez 155, Jose Guzman-Parra 156, Steven P Hamilton 157, Marian L Hamshere 136, Annette Hartmann 149, Joanna Hauser 158, Stefanie Heilmann-Heimbach 134, Per Hoffmann 132, 133, 134, Marcus Ising 159, Ian Jones 136, Lisa A Jones 160, Lina Jonsson 161, René S Kahn 2, 162, John R Kelsoe 120, 163, Kenneth S Kendler 115, Stefan Kloiber 54, 159, 164, Karestan C Koenen 165, 166, 167, Manolis Kogevinas 168, Bettina Konte 149, Marie-Odile Krebs 128, 129, 130, Mikael Landén 161, 169, Jacob Lawrence 170, Marion Leboyer 108, 171, 172, Phil H Lee 28, 29, 173, Douglas F Levinson 174, Calwing Liao 175, 176, Jolanta Lissowska 177, Susanne Lucae 159, Fermin Mayoral 156, Susan L McElroy 178, Patrick McGrath 179, Peter McGuffin 7, Andrew McQuillin 103, Sarah E Medland 180, Divya Mehta 181, 182, Ingrid Melle 92, 183, Yuri Milaneschi 184, Philip B Mitchell 151, Esther Molina 185, Gunnar Morken 186, 187, Preben Bo Mortensen 16, 81, 96, 188, Bertram Müller-Myhsok 116, 189, 190, Caroline Nievergelt 120, Vishwajit Nimgaonkar 191, Markus M Nöthen 134, Michael C O'Donovan 136, Roel A Ophoff 192, 193, Michael J Owen 136, Carlos Pato 194, 195, Michele T Pato 195, Brenda WJH Penninx 184, 184, Jonathan Pimm 103, Giorgio Pistis 126, James B Potash 196, Robert A Power 7, 197, 198, Martin Preisig 126, Digby Quested 199, Josep Antoni Ramos-Quiroga 97, 98, 100, 119, Andreas Reif 200, Marta Ribasés 97, 98, 99, 100, Vanesa Richarte 97, 98, 119, Marcella Rietschel 201, Margarita Rivera 7, 202, Andrea Roberts 203, Gloria Roberts 151, Guy A Rouleau 176, 204, Diego L Rovaris 205, Dan Rujescu 149, Cristina Sánchez-Mora 97, 98, 99, 100, Alan R Sanders 147, 148, Peter R Schofield 145, 146, Thomas G Schulze 122, 142, 206, 207, 208, Laura J Scott 117, Alessandro Serretti 209, Jianxin Shi 210, Stanley I Shyn 211, Lea Sirignano 142, Pamela Sklar 1, 2, 212, Olav B Smeland 92, 93, Jordan W Smoller 28, 167, 213, Edmund J S Sonuga-Barke 214, Gianfranco Spalletta 215, 216, John S Strauss 54, 164, Beata Świątkowska 217, Maciej Trzaskowski 123, Gustavo Turecki 218, Laura Vilar-Ribó 97, 100, John B Vincent 219, Henry Völzke 220, James TR Walters 136, Cynthia Shannon Weickert 145, 151, Thomas W Weickert 145, 151, Myrna M Weissman 221, 222, Leanne M Williams 223, Naomi R Wray 123, 182, Clement C Zai 28, 164, 166, 224, 225, 226, Allison E. Ashley-Koch 227, Jean C. Beckham 228, 229, Elizabeth R. Hauser 227, 230, Michael A. Hauser 227, Nathan A. Kimbrel 228, 229, Jennifer H. Lindquist 231, Benjamin McMahon 232, David W. Oslin 233, 234, Xuejun Qin 227, Major Depressive Disorder Working Group of the Psychiatric Genomics Consortium, Bipolar Disorder Working Group of the Psychiatric Genomics Consortium, Eating Disorders Working Group of the Psychiatric Genomics Consortium, German Borderline Genomics Consortium, MVP Suicide Exemplar Workgroup, VA Million Veteran Program, Esben Agerbo 81, 188, 235, Anders D Børglum 15, 16, 17, 18, Gerome Breen 6, 7, Annette Erlangsen 18, 236, 237, 238, Tõnu Esko 239, 240, Joel Gelernter 11, 12, David M Hougaard 235, 241, Ronald C Kessler 242, Henry R Kranzler 243, 244, Qingqin S Li 245, Nicholas G Martin 246, Andrew M McIntosh 23, Ole Mors 235, 247, Merete Nordentoft 235, 248, Catherine M Olsen 249, David Porteous 250, Robert J Ursano 251, Danuta Wasserman 72, Thomas Werge 95, 235, 252, 253, David C Whiteman 249, Cynthia M Bulik 22, 169, 254, Hilary Coon 14, 255, Ditte Demontis 15, 16, 17, 18, Anna R Docherty 8, 14, Po-Hsiu Kuo 19, 39, Cathryn M Lewis 7, 256, J John Mann 257, Miguel E Rentería 4, 5, Daniel J Smith 258, Eli A Stahl 1, 2, 239, Murray B Stein 259, Fabian Streit 78, Virginia Willour 260, Douglas M Ruderfer 3, 261, 262

**Affiliations**

1, Department of Genetics and Genomic Sciences, Icahn School of Medicine at Mount Sinai, New York, NY, US

2, Department of Psychiatry, Icahn School of Medicine at Mount Sinai, New York, NY, US

3, Division of Genetic Medicine, Department of Medicine, Vanderbilt Genetics Institute, Vanderbilt University Medical Center, Nashville, TN, US

4, Department of Genetics and Computational Biology, QIMR Berghofer Medical Research Institute, Brisbane, QLD, Australia

5, School of Biomedical Sciences, Faculty of Medicine, The University of Queensland, Brisbane, QLD, Australia

6, National Institute for Health Research (NIHR) Maudsley Biomedical Research Centre at South London and Maudsley NHS Foundation Trust, King's College London, London, UK

7, Social Genetic and Developmental Psychiatry Centre, King's College London, London, UK

8, Department of Psychiatry, Virginia Commonwealth University, Richmond, VA, US

9, Department of Biostatistics, Columbia University, New York, NY, US

10, Department of Psychiatry, Columbia University, New York, NY, US

11, Department of Psychiatry, Veterans Affairs Connecticut Healthcare Center, West Haven, CT, US

12, Division of Human Genetics, Department of Psychiatry, Yale University School of Medicine, New Haven, CT, US

13, Department of Psychiatry and Behavioral Sciences, Emory University School of Medicine, Atlanta, GA, US

14, Department of Psychiatry, University of Utah School of Medicine, Salt Lake City, UT, US

15, Centre for Genomics and Personalized Medicine, CGPM, Aarhus University, Aarhus, Denmark

16, Centre for Integrative Sequencing, iSEQ, Aarhus University, Aarhus, Denmark

17, Department of Biomedicine, Aarhus University, Aarhus, Denmark

18, The Lundbeck Foundation Initiative for Integrative Psychiatric Research, iPSYCH, Aarhus University, Aarhus, Denmark

19, Institute of Epidemiology and Preventive Medicine, College of Public Health, National Taiwan University, Taipei, Taiwan

20, School of Psychology, Curtin University, Perth, Western Australia, Australia

21, Division of Paediatrics, The University of Western Australia, Perth, Western Australia, Australia

22, Department of Psychiatry, University of North Carolina at Chapel Hill, Chapel Hill, NC, US

23, Division of Psychiatry, University of Edinburgh, Edinburgh, UK

24, Department of Psychiatry and Psychotherapy, Charité - Universitätsmedizin Berlin, Berlin, Germany

25, Department of Psychiatry and Biobehavioral Science, Semel Institute, David Geffen School of Medicine, University of California, Los Angeles, Los Angeles, CA, US

26, Department of Psychiatry, Yokohama City University Graduate School of Medicine, Yokohama, Japan

27, Department of Psychiatry, Kobe University Graduate School of Medicine, Kobe, Japan

28, Stanley Center for Psychiatric Research, Broad Institute, Cambridge, MA, US

29, Analytical and Translational Genetics Unit, Massachusetts General Hospital, Boston, MA, US

30, Population Studies Center, Institute for Social Research, University of Michigan, Ann Arbor, MI, US

31, Survery Research Center, Institute for Social Research, University of Michigan, Ann Arbor, MI, US

32, BioRealm, LLC, Walnut, CA, US

33, Oregon Research Institute, Eugene, OR, US

34, Department of Psychiatry, Center for Neurobiology and Behavior, Perelman School of Medicine at the University of Pennsylvania, Philadelphia, PA, US

35, Department of Psychosomatic Medicine and Psychotherapy, Central Institute of Mental Health, Medical Faculty Mannheim, University of Heidelberg, Mannheim, Germany

36, The Center for Eating Disorders at Sheppard Pratt, Baltimore, MD, US

37, Center for Applied Genomics, Children's Hospital of Philadelphia, Philadelphia, PA, US

38, Center for Neuropsychiatric Research, National Health Research Institutes, Miaoli County, Taiwan

39, Department of Psychiatry, National Taiwan University Hospital, Taipei, Taiwan

40, Department of Psychiatry, University of Minnesota, Minneapolis, MN, US

41, Hôpital Sainte Anne, GHU Paris Psychiatrie et Neurosciences, Paris, France

42, Institute of Psychiatry and Neuroscience of Paris (IPNP), INSERM U1266, Université de Paris, Paris, France

43, Department of Psychiatry, University Hospital Bellvitge-IDIBELL and CIBEROBN, Barcelona, Spain

44, Department of Psychiatry and Psychotherapy, Ludwig-Maximilians-University (LMU), Munich, Germany

45, Schön Klinik Roseneck affiliated with the Medical Faculty of the University of Munich (LMU), Munich, Germany

46, Department of Surgery, Faculty of Medicine, University of Toronto, Toronto, Canada

47, Department of Psychiatry and Behavioral Sciences, SUNY Upstate Medical University, Syracuse, NY, US

48, The Perelman School of Medicine, University of Pennsylvania, Philadelphia, PA, US

49, Department of Psychiatry, Weill Cornell Medical College, New York, NY, US

50, Department of Psychiatry, National Taiwan University Hospital and College of Medicine, Taipei, Taiwan

51, Biostatistics Research Center, Herbert Wertheim School of Public Health and Human Longevity Science, University of California San Diego, La Jolla, CA, US

52, Inserm U955, Institut Mondor de recherches Biomédicales, Laboratoire, Neuro-Psychiatrie Translationnelle, and Fédération Hospitalo-Universitaire de Précision Médecine en Addictologie et Psychiatrie (FHU ADAPT), University Paris-Est-Créteil, Créteil, France

53, Eating Recovery Center, Denver, CO, US

54, Centre for Addiction and Mental Health, Toronto, ON, Canada

55, Department of Psychiatry, University of Toronto, Toronto, Canada

56, Institute of Medical Science, University of Toronto, Toronto, Canada

57, Department of Psychiatry, University of California San Diego, San Diego, CA, US

58, Department of Psychology, Florida State University, Tallahassee, FL, US

59, Department of Psychology, Michigan State University, Lansing, MI, US

60, Department of Psychiatry and Psychotherapy, University Medical Center, Mainz, Germany

61, Department of Clinical Psychology, The Chicago School of Professional Psychology, Washington DC, Washington, DC, US

62, BESE Division, King Abdullah University of Science and Technology, Thuwal, Saudi Arabia

63, Department of Psychiatry, University of Lausanne-University Hospital of Lausanne (UNIL-CHUV), Lausanne, Switzerland

64, Department of Paediatric Laboratory Medicine, The Hospital for Sick Children, Toronto, Canada

65, Department of Psychiatry and Behavioral Science, University of North Dakota School of Medicine and Health Sciences, Fargo, ND, US

66, HudsonAlpha Institute for Biotechnology, Huntsville, AL, US

67, Department of Psychiatry, Charité - Universitätsmedizin Berlin, Corporate Member of Freie Universität Berlin, Humboldt-Universität zu Berlin, Berlin Institute of Health, Campus Benjamin Franklin, Berlin, Germany

68, Department of Psychiatry, Neurobiology, Pharmacology, and Biotechnologies, University of Pisa, Pisa, Italy

69, Department of Psychology, Saint-Petersburg State University, Saint-Petersburg, Russian Federation

70, Department of Borderline Disorders and Psychotherapy, V.M. Bekhterev National Medical Research Center for Psychiatry and Neurology, Saint-Petersburg, Russian Federation

71, Department of Genetics and Genomic Biology, The Hospital for Sick Children, Toronto, Canada

72, National Centre for Suicide Research and Prevention of Mental Ill-Health (NASP), LIME, Karolinska Institutet, Stockholm, Sweden

73, David Geffen School of Medicine, University of California Los Angeles, Los Angeles, LA, US

74, Department of Psychiatry and Biobehavioral Science, Semel Institute for Neuroscience and Human Behavior, University of California Los Angeles, Los Angeles, LA, US

75, Institute of Psychiatry, Psychology and Neuroscience, Department of Psychological Medicine, King’s College London, London, UK

76, National Institute for Health Research Biomedical Research Centre, King’s College London and South London and Maudsley National Health Service Foundation Trust, London, UK

77, Center for Behavioral Genomics, Department of Psychiatry, University of California, San Diego, San Diego, CA, US

78, Department of Genetic Epidemiology in Psychiatry, Central Institute of Mental Health, Medical Faculty Mannheim, University of Heidelberg, Mannheim, Germany

79, Centre for Mental Health, University Health Network, Toronto, Canada

80, Program for Eating Disorders, University Health Network, Toronto, Canada

81, National Centre for Register-Based Research, Aarhus University, Aarhus, Denmark

82, Department of Genetics, University of North Carolina at Chapel Hill, Chapel Hill, NC, US

83, Department of Clinical Sciences, Psychiatry, Umeå University Medical Faculty, Umeå, Sweden

84, Department of Psychiatric Research, Diakonhjemmet Hospital, Oslo, Norway

85, Department of Clinical Neuroscience, Centre for Psychiatry Research, Karolinska Institutet, Stockholm, Sweden

86, NORMENT, Institute of Clinical Medicine, University of Oslo, Oslo, Norway

87, Discipline of Psychiatry, University of Adelaide, Adelaide, SA, Australia

88, Department of Psychiatry, Dalhousie University, Halifax, NS, Canada

89, National Institute of Mental Health, Klecany, CZ

90, Department of Clinical Neuroscience, Karolinska Institutet, Stockholm, Sweden

91, Inst of Environmental Medicine, Karolinska Institutet, Stockholm, Sweden

92, Division of Mental Health and Addiction, Oslo University Hospital, Oslo, Norway

93, NORMENT, University of Oslo, Oslo, Norway

94, Psychiatry, Berkshire Healthcare NHS Foundation Trust, Bracknell, UK

95, Institute of Biological Psychiatry, Copenhagen Mental Health Services, Copenhagen University Hospital, Copenhagen, Denmark

96, The Lundbeck Foundation Initiative for Integrative Psychiatric Research, iPSYCH, Copenhagen, Denmark

97, Department of Psychiatry, Hospital Universitari Vall d’Hebron, Barcelona, Spain

98, Biomedical Network Research Centre on Mental Health (CIBERSAM), Instituto de Salud Carlos III, Madrid, Spain

99, Department of Genetics, Microbiology & Statistics, University of Barcelona, Barcelona, Spain

100, Psychiatric Genetics Unit, Group of Psychiatry, Mental Health and Addiction, Vall d’Hebron Research Institute (VHIR), Universitat Autònoma de Barcelona, Barcelona, Spain

101, Department of Psychiatry and Psychotherapy, University Medicine Greifswald, Greifswald, Mecklenburg-Vorpommern, Germany

102, Department of Psychiatry, University of Coimbra, Coimbra, Portugal

103, Division of Psychiatry, University College London, London, UK

104, Laboratory of Developmental Psychiatry, Hospital de Clínicas de Porto Alegre, Porto Alegre, RS, Brazil

105, Department of Genetics, Universidade Federal do Rio Grande do Sul, Porto Alegre, RS, Brazil

106, Department of Psychiatry, Melbourne Medical School, University of Melbourne, Melbourne, Australia

107, Department of Psychiatry, University of Münster, Münster, Germany

108, Department of Psychiatry and Addiction Medicine, Assistance Publique - Hôpitaux de Paris, Paris, France

109, Paris Bipolar and TRD Expert Centres, FondaMental Foundation, Paris, France

110, UMR-S1144 Team 1: Biomarkers of relapse and therapeutic response in addiction and mood disorders, INSERM, Paris, France

111, Psychiatry, Université Paris Diderot, Paris, France

112, Institute of Epidemiology and Social Medicine, University of Münster, Münster, Nordrhein-Westfalen, Germany

113, Health Sciences Research, Mayo Clinic, Rochester, MN, US

114, Department of Psychiatry and Behavioral Sciences, State University of New York Downstate Medical Center, New York, NY, US

115, Department of Psychiatry, Virginia Commonwealth University, Richmond, VA, US

116, Department of Translational Research in Psychiatry, Max Planck Institute of Psychiatry, Munich, Germany

117, Center for Statistical Genetics and Department of Biostatistics, University of Michigan, Ann Arbor, MI, US

118, Psychiatry, UMC Utrecht Hersencentrum, Utrecht, Netherlands

119, Department of Psychiatry and Legal Medicine, Universitat Autònoma de Barcelona, Barcelona, Spain

120, Department of Psychiatry, University of California San Diego, La Jolla, CA, US

121, School of Psychology, University of New South Wales, Sydney, NSW, Australia

122, Institute of Psychiatric Phenomics and Genomics (IPPG), University Hospital, LMU Munich, Munich, Germany

123, Institute for Molecular Bioscience, The University of Queensland, Brisbane, QLD, Australia

124, Centre for Children’s Health Research, The University of Queensland, Brisbane, QLD, Australia

125, Department of Psychiatry, UMC Utrecht Hersencentrum Rudolf Magnus, Utrecht, Netherlands

126, Department of Psychiatry, Lausanne University Hospital and University of Lausanne, Lausanne, Vaud, Switzerland

127, Mental Health Unit, Department of Psychiatry, Faculty of Medicine, Granada University Hospital Complex, University of Granada, Granada, Spain

128, Institut de Psychiatrie, CNRS GDR 3557, Paris, France

129, Department of Evaluation, Prevention and Therapeutic innovation, GHU Paris Psychiatrie et Neurosciences, Paris, France

130, Team Pathophysiology of psychiatric diseases, Université de Paris, Institute of Psychiatry and Neuroscience of Paris (IPNP), INSERM U1266, Paris, France

131, Institute of Neuroscience and Medicine (INM-1), Research Centre Jülich, Jülich, Germany

132, Institute of Medical Genetics and Pathology, University Hospital Basel, Basel, Switzerland

133, Department of Biomedicine, University of Basel, Basel, Switzerland

134, Institute of Human Genetics, University of Bonn, School of Medicine & University Hospital Bonn, Bonn, Germany

135, Neuropsychiatric Genetics Research Group, Dept of Psychiatry and Trinity Translational Medicine Institute, Trinity College Dublin, Dublin, Ireland

136, Medical Research Council Centre for Neuropsychiatric Genetics and Genomics, Division of Psychological Medicine and Clinical Neurosciences, Cardiff University, Cardiff, UK

137, Department of Translational Genomics, University of Southern California, Pasadena, CA, US

138, Department of Medical Genetics, Oslo University Hospital, Oslo, Norway

139, NORMENT, KG Jebsen Centre for Psychosis Research, Department of Clinical Science, University of Bergen, Bergen, Norway

140, Department of Medical & Molecular Genetics, Indiana University, Indianapolis, IN, US

141, Biochemistry and Molecular Biology, Indiana University School of Medicine, Indianapolis, IN, US

142, Department of Genetic Epidemiology in Psychiatry, Central Institute of Mental Health, Medical Faculty Mannheim, Heidelberg University, Mannheim, Germany

143, Centre for Human Genetics, University of Marburg, Marburg, Germany

144, Department of Psychiatry & Psychology, Mayo Clinic, Rochester, MN, US

145, Neuroscience Research Australia, Sydney, NSW, Australia

146, School of Medical Sciences, University of New South Wales, Sydney, NSW, Australia

147, Department of Psychiatry and Behavioral Sciences, NorthShore University HealthSystem, Evanston, IL, US

148, Department of Psychiatry and Behavioral Neuroscience, University of Chicago, Chicago, IL, US

149, Dept. of Psychiatry, Psychotherapy and Psychosomatics, Martin-Luther-University Halle-Wittenberg, Halle (Saale), Germany

150, Department of Psychiatry, University of Munich, Munich, Germany

151, School of Psychiatry, University of New South Wales, Sydney, NSW, Australia

152, ADHD Outpatient Program, Adult Division, Hospital de Clínicas de Porto Alegre, Porto Alegre, RS, Brazil

153, Department of Psychiatry, Universidade Federal do Rio Grande do Sul, Porto Alegre, RS, Brazil

154, Biometric Psychiatric Genetics Research Unit, Alexandru Obregia Clinical Psychiatric Hospital, Bucharest, Romania

155, Department of Psychiatry, Faculty of Medicine and Biomedical Research Centre (CIBM), University of Granada, Granada, Spain

156, Mental Health Department, University Regional Hospital. Biomedicine Institute (IBIMA), Málaga, Spain

157, Psychiatry, Kaiser Permanente Northern California, San Francisco, CA, US

158, Department of Psychiatry, Laboratory of Psychiatric Genetics, Poznan University of Medical Sciences, Poznan, Poland

159, Max Planck Institute of Psychiatry, Munich, Germany

160, Department of Psychological Medicine, University of Worcester, Worcester, UK

161, Department of Psychiatry and Neuroscience, University of Gothenburg, Gothenburg, Sweden

162, Psychiatry, UMC Utrecht Hersencentrum Rudolf Magnus, Utrecht, Netherlands

163, Institute for Genomic Medicine, University of California San Diego, La Jolla, CA, US

164, Department of Psychiatry, University of Toronto, Toronto, ON, Canada

165, Stanley Center for Psychiatric Research, Broad Institute, Cambridge, MA, US

166, Department of Epidemiology, Harvard TH Chan School of Public Health, Boston, MA, US

167, Department of Psychiatry, Massachusetts General Hospital, Boston, MA, US

168, Center for Research in Environmental Epidemiology (CREAL), Barcelona, Spain

169, Department of Medical Epidemiology and Biostatistics, Karolinska Institutet, Stockholm, Sweden

170, Psychiatry, North East London NHS Foundation Trust, Ilford, UK

171, INSERM, Paris, France

172, Faculté de Médecine, Université Paris Est, Créteil, France

173, Psychiatric and Neurodevelopmental Genetics Unit, Massachusetts General Hospital, Boston, MA, US

174, Psychiatry & Behavioral Sciences, Stanford University, Stanford, CA, US

175, Department of Human Genetics, McGill University, Montreal, QC, Canada

176, Montreal Neurological Institute and Hospital, Montreal, QC, Canada

177, Cancer Epidemiology and Prevention, M. Sklodowska-Curie Cancer Center and Institute of Oncology, Warsaw, Poland

178, Research Institute, Lindner Center of HOPE, Mason, OH, US

179, Psychiatry, Columbia University College of Physicians and Surgeons, New York, NY, US

180, Genetics and Computational Biology, QIMR Berghofer Medical Research Institute, Brisbane, QLD, Australia

181, School of Psychology and Counseling, Queensland University of Technology, Brisbane, QLD, Australia

182, Queensland Brain Institute, The University of Queensland, Brisbane, QLD, Australia

183, Division of Mental Health and Addiction, University of Oslo, Institute of Clinical Medicine, Oslo, Norway

184, Department of Psychiatry, Amsterdam UMC, Vrije Universiteit and GGZ inGeest, Amsterdam, Netherlands

185, Department of Nursing, Faculty of Health Sciences and Biomedical Research Centre (CIBM), University of Granada, Granada, Spain

186, Mental Health, Faculty of Medicine and Health Sciences, Norwegian University of Science and Technology - NTNU, Trondheim, Norway

187, Psychiatry, St Olavs University Hospital, Trondheim, Norway

188, Centre for Integrated Register-based Research, Aarhus University, Aarhus, Denmark

189, Munich Cluster for Systems Neurology (SyNergy), Munich, Germany

190, University of Liverpool, Liverpool, UK

191, Psychiatry and Human Genetics, University of Pittsburgh, Pittsburgh, PA, US

192, Psychiatry, Erasmus University Medical Center, Rotterdam, Netherlands

193, Jane and Terry Semel Institute for Neuroscience and Human Behavior, Los Angeles, CA, US

194, College of Medicine Institute for Genomic Health, SUNY Downstate Medical Center College of Medicine, Brooklyn, NY, US

195, Institute for Genomic Health, SUNY Downstate Medical Center College of Medicine, Brooklyn, NY, US

196, Psychiatry, University of Iowa, Iowa City, IA, US

197, Genetics, BioMarin Pharmaceuticals, London, UK

198, St Edmund Hall, University of Oxford, Oxford, UK

199, Department of Psychiatry, University of Oxford, Oxford, UK

200, Department of Psychiatry, Psychosomatic Medicine and Psychotherapy, University Hospital Frankfurt, Frankfurt, Germany

201, Department of Genetic Epidemiology in Psychiatry, Central Institute of Mental Health, Medical Faculty Mannheim, Heidelberg University, Mannheim, Baden-Württemberg, Germany

202, Department of Biochemistry and Molecular Biology II and Institute of Neurosciences, Biomedical Research Centre (CIBM), University of Granada, Granada, Spain

203, Department of Environmental Health, Harvard TH Chan School of Public Health, Boston, MA, US

204, Department of Neurology and Neurosurgery, McGill University, Faculty of Medicine, Montreal, QC, Canada

205, Department of Physiology and Biophysics, Instituto de Ciencias Biomedicas Universidade de Sao Paulo, São Paulo, SP, Brazil

206, Department of Psychiatry and Behavioral Sciences, Johns Hopkins University School of Medicine, Baltimore, MD, US

207, Human Genetics Branch, Intramural Research Program, National Institute of Mental Health, Bethesda, MD, US

208, Department of Psychiatry and Psychotherapy, University Medical Center Göttingen, Göttingen, Germany

209, Department of Biomedical and NeuroMotor Sciences, University of Bologna, Bologna, Italy

210, Division of Cancer Epidemiology and Genetics, National Cancer Institute, Bethesda, MD, US

211, Behavioral Health Services, Kaiser Permanente Washington, Seattle, WA, US

212, Department of Neuroscience, Icahn School of Medicine at Mount Sinai, New York, NY, US

213, Psychiatric and Neurodevelopmental Genetics Unit (PNGU), Massachusetts General Hospital, Boston, MA, US

214, Institute of Psychology, Psychiatry & Neuroscience, King's College London, London, UK

215, Menninger Department of Psychiatry and Behavioral Sciences, Baylor College of Medicine, Houston, Houston, TX, US

216, Laboratory of Neuropsychiatry, IRCCS Santa Lucia Foundation, Rome, Rome, Italy

217, Department of Environmental Epidemiology, Nofer Institute of Occupational Medicine, Lodz, Poland

218, Department of Psychiatry, McGill University, Montreal, QC, Canada

219, Molecular Brain Science, Centre for Addiction and Mental Health, Toronto, ON, Canada

220, Institute for Community Medicine, University Medicine Greifswald, Greifswald, Mecklenburg-Vorpommern, Germany

221, Columbia University College of Physicians and Surgeons, New York, NY, US

222, Division of Translational Epidemiology, New York State Psychiatric Institute, New York, NY, US

223, Department of Psychiatry and Behavioral Sciences, Stanford University, Stanford, CA, US

224, Institute of Medical Science, University of Toronto, Toronto, ON, Canada

225, Molecular Brain Science, Campbell Family Mental Health Research Institute, Centre for Addiction and Mental Health, Toronto, ON, Canada

226, Laboratory Medicine and Pathobiology, University of Toronto, Toronto, ON, Canada

227, Duke Molecular Physiology Institute, Duke University Medical Center, Durham, NC, USA

228, VISN 6 Mid-Atlantic Mental Illness Research, Education, and Clinical Center, Durham Veterans Affairs Health Care System, Durham, NC, USA

229, Department of Psychiatry and Behavioral Sciences, Duke University School of Medicine, Durham, NC, USA

230, Cooperative Studies Program Epidemiology Center, Durham Veterans Affairs Health Care System, Durham, NC, USA

231, VA Health Services Research and Development Center of Innovation to Accelerate Discovery and Practice Transformation, Durham Veterans Affairs Health Care System, Durham, NC, USA

232, Theoretical Division, Los Alamos National Laboratory, Los Alamos National Laboratory, Los Alamos, NM, USA

233, VISN 4 Mental Illness Research, Education, and Clinical Center, Corporal Michael J. Crescenz VA Medical Center, Philadelphia, PA, USA

234, Department of Psychiatry, Perelman School of Medicine, University of Pennsylvania, Philadelphia, PA, USA

235, The Lundbeck Foundation Initiative for Integrative Psychiatric Research, iPSYCH, Aarhus, Denmark

236, Center of Mental Health Research, Australian National University, Canberra, Australia

237, Department of Mental Health, Johns Hopkins Bloomberg School of Public Health, Baltimore, MD, US

238, Danish Research Institute for Suicide Prevention, Mental Health Centre Copenhagen, Copenhagen, Denmark

239, Program in Medical and Population Genetics, Broad Institute, Cambridge, MA, US

240, Estonian Genome Center, Institute of Genomics, University of Tartu, Tartu, Estonia

241, Center for Neonatal Screening, Department for Congenital Disorders, Statens Serum Institut, Copenhagen, Denmark

242, Department of Health Care Policy, Harvard Medical School, Boston, MA, US

243, Department of Psychiatry, University of Pennsylvania Perelman School of Medicine, Philadelphia, PA, US

244, VISN 4 MIRECC, Crescenz VAMC, Philadelphia, PA, US

245, Neuroscience, Janssen Research & Development, LLC, Titusville, NJ, US

246, Department of Genetics and Computational Biology, QIMR Berghofer Medical Research Institute, Herston, QLD, Australia

247, Psychosis Research Unit, Aarhus University Hospital, Risskov, Aarhus, Denmark

248, Mental Health Center Copenhagen, Copenhagen University Hospital, Copenhagen, Denmark

249, Department of Population Health, QIMR Berghofer Medical Research Institute, Herston, QLD, Australia

250, Institute for Genetics and Molecular Medicine, University of Edinburgh, Edinburgh, UK

251, Department of Psychiatry, Uniformed University of the Health Sciences, Bethesda, MD, US

252, Department of Clinical Medicine, University of Copenhagen, Copenhagen, Denmark

253, Lundbeck Foundation GeoGenetics Centre, GLOBE Institute, University of Copenhagen, Copenhagen, Denmark

254, Department of Nutrition, University of North Carolina at Chapel Hill, Chapel Hill, NC, US

255, Biomedical Informatics, University of Utah School of Medicine, Salt Lake City, UT, US

256, Department of Medical & Molecular Genetics, King's College London, London, UK

257, Departments of Psychiatry and Radiology, Columbia University, New York, NY, US

258, Institute of Health and Wellbeing, University of Glasgow, Glasgow, UK

259, Department of Psychiatry and School of Public Health, University of California San Diego, La Jolla, CA, US

260, Department of Psychiatry, University of Iowa, Iowa City, IA, US

261, Department of Biomedical Informatics, Vanderbilt University Medical Center, Nashville, TN, US

262, Department of Psychiatry and Behavioral Sciences, Vanderbilt University Medical Center, Nashville, TN, US
