# Supplementary material for: Genome-wide association study identifies four pan-ancestry loci for suicidal ideation in the Million Veteran Program
Source: PLoS Genet. 2023 Mar 20;19(3):e1010623. doi: 10.1371/journal.pgen.1010623 (PMC10063168; doi:10.1371/journal.pgen.1010623)
Supplement: S1 Table — (DOCX) [file pgen.1010623.s005.docx]

**Supplementary Table 1. ICD9 and ICD10 Codes Used to Phenotype Suicidal Ideation and Suicide Attempts.**

| **Phenotype Category** | **Code Type** | **ICD Code** | **ICD Description** |
| --- | --- | --- | --- |
| Attempt^a^ | ICD10 | T14.91 | Suicide attempt |
| Attempt^a^ | ICD10 | X60-X69 | Intentional self-poisoning |
| Attempt^a^ | ICD10 | X70 -X83 | Intentional self-harm |
| Attempt^a^ | ICD10 | Y87.0 | Sequelae of intentional self-harm |
| Attempt^a^ | ICD10 | Z91.5 | Personal history of self-harm |
| Attempt^a^ | ICD9 | E950.- E959 | Suicide and self-inflicted poisoning |
| Ideation | ICD10 | R45.851 | Suicidal ideations |
| Ideation | ICD9 | V62.84 | Suicidal ideation |
| Exclude^b^ | ICD10 | F00-F99 | Mental, Behavioral and Neurodevelopmental disorders |
| Exclude^b^ | ICD10 | R53 | Fatigue |
| Exclude^b^ | ICD10 | R628 | Other lack of expected normal physiological development |
| Exclude^b^ | ICD10 | R96 | Other sudden death, cause unknown |
| Exclude^b^ | ICD10 | R99 | Ill-defined and unknown cause of mortality |
| Exclude^b^ | ICD10 | W00-W19 | Slipping, tripping, stumbling and falls |
| Exclude^b^ | ICD10 | W20-W49 | Exposure to inanimate mechanical forces |
| Exclude^b^ | ICD10 | W50-W64 | Exposure to animate mechanical forces |
| Exclude^b^ | ICD10 | W65-W74 | Accidental non-transport drowning and submersion |
| Exclude^b^ | ICD10 | W85-W99 | Exposure to electric current, radiation and extreme ambient air temperature and pressure |
| Exclude^b^ | ICD10 | X00-X08 | Exposure to smoke, fire, and flames |
| Exclude^b^ | ICD10 | X10-X19 | Contact with heat and hot substances |
| Exclude^b^ | ICD10 | X30-X39 | Exposure to forces of nature |
| Exclude^b^ | ICD10 | X50-X50 | Overexertion and strenuous or repetitive movements |
| Exclude^b^ | ICD10 | X52-X59 | Accidental exposure to other specified factors |
| Exclude^b^ | ICD10 | Y10-Y19 | Poisoning, undetermined intent |
| Exclude^b^ | ICD10 | Y20-Y21 | Hanging, Drowning, undetermined intent |
| Exclude^b^ | ICD10 | Y22-Y24 | Firearm discharge, undetermined intent |
| Exclude^b^ | ICD10 | Y25-Y29 | Contact with dangerous elements, undetermined intent |
| Exclude^b^ | ICD10 | Y30-Y34 | Falling, jumping, crashing, undetermined intent |
| Exclude^b^ | ICD10 | Y86 | Sequelae of other accidents |
| Exclude^b^ | ICD10 | Y872 | Event of undetermined intent |

*Note: ^a^Veterans who had a history of suicide attempts were excluded from the present analyses. ^b^Suspicious/Indeterminate Cause of death record in NDI*
