# Supplementary material for: Genome-wide association study identifies four pan-ancestry loci for suicidal ideation in the Million Veteran Program
Source: PLoS Genet. 2023 Mar 20;19(3):e1010623. doi: 10.1371/journal.pgen.1010623 (PMC10063168; doi:10.1371/journal.pgen.1010623)
Supplement: S2 Table — (DOCX) [file pgen.1010623.s006.docx]

**Supplementary Table 2. Suicide Prevention Applications Network (SPAN) Codes Used to Phenotype Suicide Attempts and Suicidal Ideation.**

| **Phenotype Category** | **Code Type*** | **Description** |
| --- | --- | --- |
| Attempt^a^ | E-Type | 1=Attempt (Non-fatal Suicidal Attempt) |
| Attempt^a^ | E-Type | 2=Completion (Fatal Suicidal Attempt) |
| Attempt^a^ | SDV Subclass | 7 - Suicide Attempt; Without Injury |
| Attempt^a^ | SDV Subclass | 10 - Suicide Attempt; Without Injury; Interrupted by Self/Other |
| Attempt^a^ | SDV Subclass | 16 - Suicide Attempt; With Injury |
| Attempt^a^ | SDV Subclass | 19 - Suicide Attempt; With Injury; Interrupted by Self/Other |
| Attempt^a^ | SDV Subclass | 22 - Suicide |
| Ideation | E-Type | 4=Ideation |
| Ideation | SDV Subclass | 2 - Suicidal Ideation; With Undetermined Suicidal Intent |
| Ideation | SDV Subclass | 3 - Suicidal Ideation; Without Suicidal Intent |
| Ideation | SDV Subclass | 4 - Suicidal Ideation; With Suicidal Intent |
| Ideation | SDV Subclass | 13 - Suicidal SDV; Preparatory |
| Exclude^b^ | SDV Subclass | 0=Insufficient evidence to suggest self-directed violence |
| Exclude^b^ | SDV Subclass | 1=Non-Suicidal SDV Ideation |
| Exclude^b^ | SDV Subclass | 5=Undetermined SDV; Without Injury |
| Exclude^b^ | SDV Subclass | 6=Non-Suicidal SDV; Without Injury |
| Exclude^b^ | SDV Subclass | 8=Undetermined SDV; Without Injury; Interrupted by Self/Other |
| Exclude^b^ | SDV Subclass | 9=Non-Suicidal SDV; Without Injury; Interrupted by Self/Other |
| Exclude^b^ | SDV Subclass | 11=Undetermined SDV; Preparatory |
| Exclude^b^ | SDV Subclass | 12 =Non-Suicidal SDV; Preparatory |
| Exclude^b^ | SDV Subclass | 14=Undetermined SDV; With Injury |
| Exclude^b^ | SDV Subclass | 15=Non-Suicidal SDV; With Injury |
| Exclude^b^ | SDV Subclass | 17=Undetermined SDV; With Injury; Interrupted by Self/Others |
| Exclude^b^ | SDV Subclass | 18=Non-Suicidal SDV; With Injury; Interrupted by Self/Others |
| Exclude^b^ | SDV Subclass | 20=Undetermined SDV; Fatal |
| Exclude^b^ | SDV Subclass | 21=Non-Suicidal SDV; Fatal |
| Exclude^b^ | E-Type | 3=Undetermined |
| Exclude^b^ |  | If both subclass and e_type are missing, then exclude |

*Notes:* SDV = Self-directed violence; *A period existed where both the new and old classification systems overlapped. If there was a discrepancy between the old system (e-type) and the new (sdvsubclass), the 23 level sdvsubclass variable took priority. ^a^ Veterans who had a history of suicide attempts were excluded from the analyses. ^b^Veterans who had other types of entries in SPAN (e.g., nonsuicidal self-injury, self-injury with unclear intent), but had no indication of confirmed attempt or ideation from any other source were also excluded from the present analyses.
