# Supplementary material for: Genome-wide association study identifies four pan-ancestry loci for suicidal ideation in the Million Veteran Program
Source: PLoS Genet. 2023 Mar 20;19(3):e1010623. doi: 10.1371/journal.pgen.1010623 (PMC10063168; doi:10.1371/journal.pgen.1010623)
Supplement: S3 Table — (DOCX) [file pgen.1010623.s007.docx]

**Supplementary Table 3. Mental Health Survey Items Used to Phenotype Suicide Attempts and Suicidal Ideation.**

| **Phenotype Category** | **Survey Name** | **Survey Question Text** | **Survey Choice Text** |
| --- | --- | --- | --- |
| Attempt^a^ | Alcohol Use Inventory (Revised) | Have you ever attempted suicide when drinking? | Once |
| Attempt^a^ | Millon Clinical Multiaxial Inventory-III | I have tried to commit suicide. | True |
| Attempt^a^ | Millon Clinical Multiaxial Inventory-IV | I have tried to commit suicide. | True |
| Attempt^a^ | Minnesota Multiphasic Personality Inventory-2-Restructed form | I have tried to commit suicide. | True |
| Ideation | Beck Depression Inventory--Second Edition | Suicidal thoughts or wishes | I have thoughts of killing myself, but I would not carry them out. |
| Ideation | Beck Depression Inventory--Second Edition | Suicidal thoughts or wishes | I would kill myself if I had the chance. |
| Ideation | Beck Depression Inventory--Second Edition | Suicidal thoughts or wishes | I would like to kill myself. |
| Ideation | Columbia-Suicide Severity Rating Scale | Over the past month, have you had any actual thoughts of killing yourself? | Yes |
| Ideation | Columbia-Suicide Severity Rating Scale | Over the past month, have you had any actual thoughts of killing yourself? | Severe (3 to 8 hours per 24 hours) |
| Ideation | Millon Behavioral Medicine Diagnostic | I have been having serious thoughts about suicide. | True |
| Ideation | Millon Clinical Multiaxial Inventory-II | Serious thoughts of suicide have occurred to me for many years. | True |
| Ideation | Mississippi Scale | Lately, I have felt like killing myself. | Extremely True |
| Ideation | Mississippi Scale | Lately, I have felt like killing myself. | Very True |
| Ideation | Mississippi Scale | Lately, I have felt like killing myself. | Somewhat True |
| Ideation | Mississippi Scale | Lately, I have felt like killing myself. | Slightly True |
| Ideation | Minnesota Multiphasic Personality Inventory 2 | Lately I have thought a lot about killing myself. | True |
| Ideation | Minnesota Multiphasic Personality Inventory-2-Restructured Form | I have recently considered killing myself. | True |
| Ideation | Personality Assessment Inventory | I've recently been thinking about suicide. | Very True |
| Ideation | Personality Assessment Inventory | I've recently been thinking about suicide. | Mainly True |
| Ideation | Personality Assessment Inventory | I've recently been thinking about suicide. | Slightly True |
| Ideation | Personality Assessment Inventory | I have thought about suicide for a long time. | Mainly True |
| Ideation | Personality Assessment Inventory | I have thought about suicide for a long time. | Slightly True |
| Ideation | Personality Assessment Inventory | I have thought about suicide for a long time. | Very True |
| Ideation | Personality Assessment Inventory | I've thought about what I would say in a suicide note. | Mainly True |
| Ideation | Personality Assessment Inventory | I've thought about what I would say in a suicide note. | Slightly True |
| Ideation | Personality Assessment Inventory | I've thought about what I would say in a suicide note. | Very True |
| Ideation | Personality Assessment Inventory | I'm considering suicide. | Mainly True |
| Ideation | Personality Assessment Inventory | I'm considering suicide. | Very True |
| Ideation | Personality Assessment Inventory | I'm considering suicide. | Slightly True |
| Ideation | PTSD Checklist Stressor Specific | During the last two weeks have you had thoughts that you would be better off dead, or of hurting yourself in some way? | Yes |
| Ideation | PCL-5 | Thoughts that you would be better off dead or hurting yourself in some way | Several days |
| Ideation | Patient Health Questionnaire- 2 +Item9 (PHQ-2+I9) | Thoughts that you would be better off dead or hurting yourself in some way | More than half the days |
| Ideation | Patient Health Questionnaire- 2 +Item9 (PHQ-2+I9) | Thoughts that you would be better off dead or hurting yourself in some way | Nearly every day |
| Ideation | Patient Health Questionnaire- 2 +Item9 (PHQ-2+I9) | Thoughts that you would be better off dead or hurting yourself in some way | Several days |
| Ideation | Suicide Behavior Report | Is or was there evidence of suicidal intent? | Yes |

^a^ Veterans who had a history of suicidal attempts, were excluded from the present analyses.
