# Supplementary material for: Genome-wide association study identifies four pan-ancestry loci for suicidal ideation in the Million Veteran Program
Source: PLoS Genet. 2023 Mar 20;19(3):e1010623. doi: 10.1371/journal.pgen.1010623 (PMC10063168; doi:10.1371/journal.pgen.1010623)
Supplement: S4 Table — (DOCX) [file pgen.1010623.s008.docx]

**Supplementary Table 4. Ancestry-specific allele frequencies for the Genome-wide Significant Loci in Table 2.**

| **SNP** | **Chromosome:**  **Position^+^** | **Alleles**  **eff/alt** | **AFR Ancestry**  **Allele Freq.** | **ASN Ancestry**  **Allele**  **Freq** | **EUR Ancestry**  **Allele**  **Freq.** | **HIS Ancestry**  **Allele**  **Freq.** | **All Ancestries**  **Alleles Freq.** |
| --- | --- | --- | --- | --- | --- | --- | --- |
| rs77641763 | 9:140265782 | T/C | 0.035 | 0.031 | 0.11 | 0.053 | 0.093 |
| rs7185007 | 16:30927509 | C/T | 0.89 | 0.84 | 0.76 | 0.70 | 0.78 |
| rs142785607 | 2:104267493 | T/G | 0.54 | 0.13 | 0.51 | 0.42 | 0.50 |
| rs6557168 | 6:152201201 | C/T | 0.70 | 0.63 | 0.37 | 0.54 | 0.45 |
| rs73581580 | 9:140251458 | A/G | 0.14 | 0.044 | 0.11 | 0.068 | 0.11 |
| rs13211166 | 6:27265940 | A/G | 0.25 | 0.033 | 0.17 | 0.15 | 0.18 |
