# Supplementary figures and images for: Genome-wide association study identifies four pan-ancestry loci for suicidal ideation in the Million Veteran Program
Source: PLoS Genet. 2023 Mar 20;19(3):e1010623. doi: 10.1371/journal.pgen.1010623 (PMC10063168; doi:10.1371/journal.pgen.1010623)

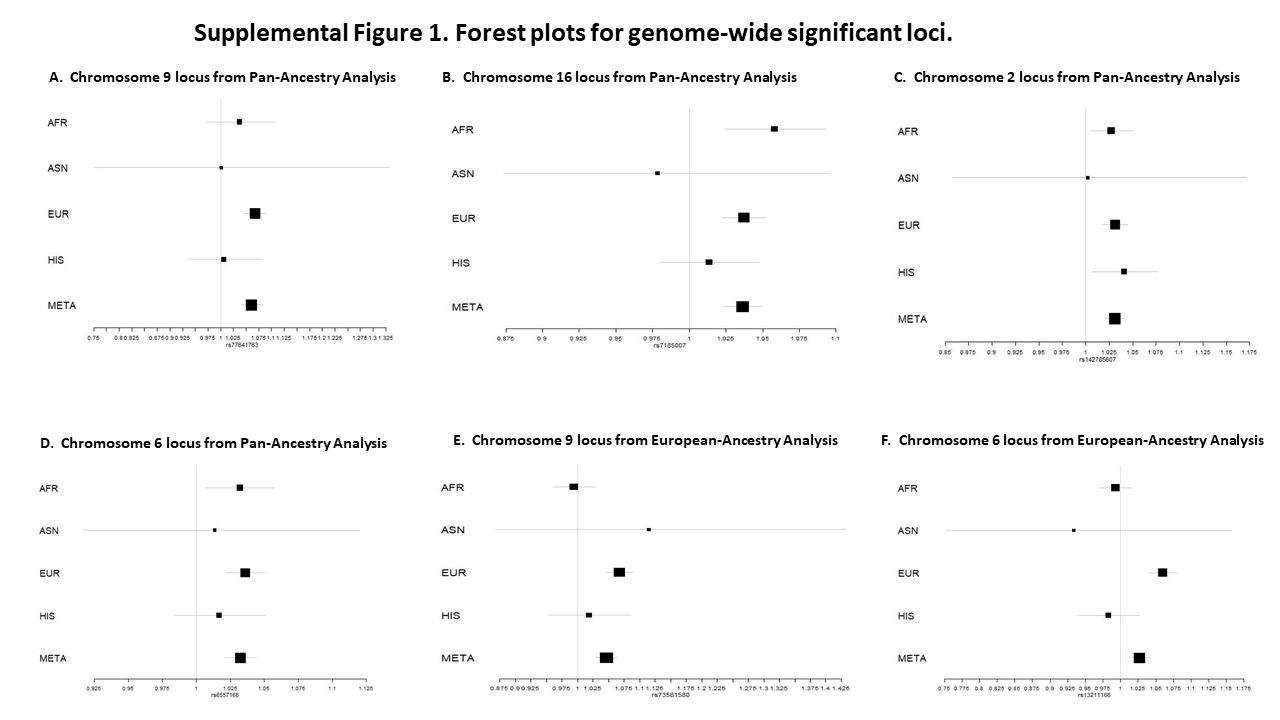

Supplement: S1 Fig — A. Chromosome 9 locus from Pan-Ancestry Analysis, B. Chromosome 16 locus from Pan-Ancestry Analysis, C. Chromosome 2 locus from Pan-Ancestry Analysis, D. Chromosome 6 locus from Pan-Ancestry Analysis, E. Chromosome 9 locus for European-Ancestry Analysis, F. Chromosome 6 locus from European-Ancestry Analysis. These plots provide the point estimates and confidence intervals at each locus by ancestry and for the meta-analysis. (TIF) [file pgen.1010623.s009.tif]

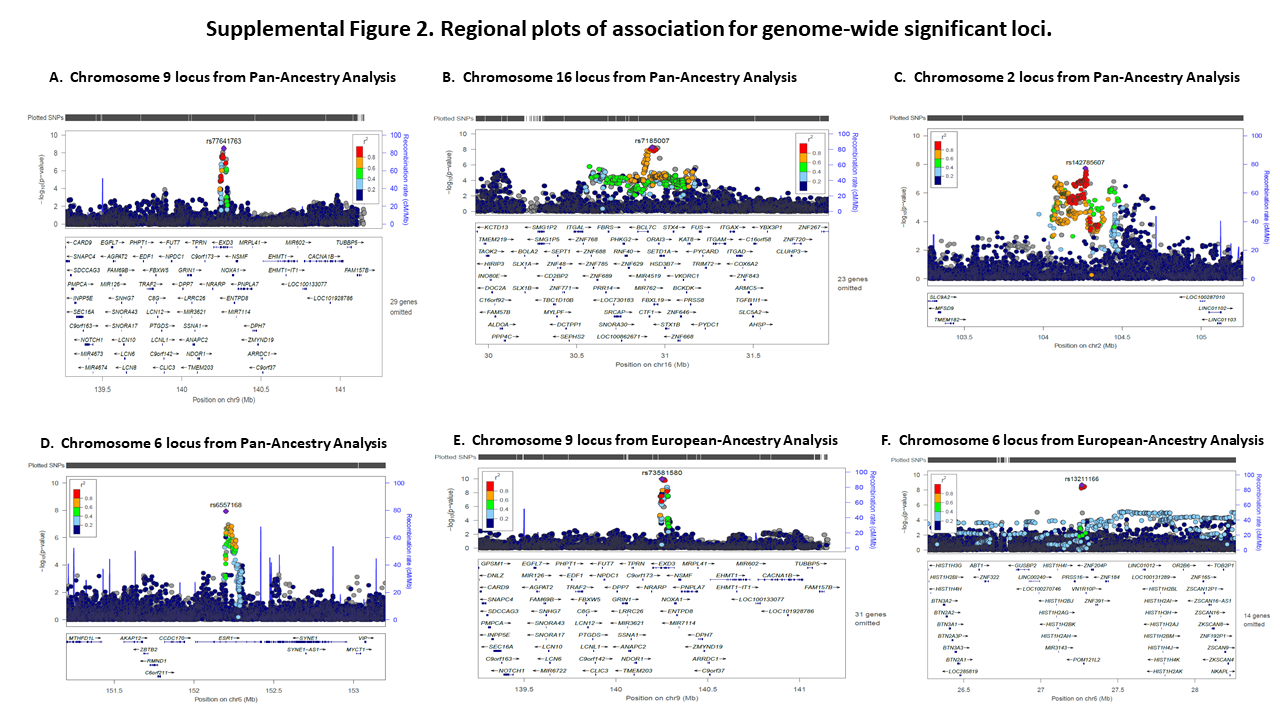

Supplement: S2 Fig — A. Chromosome 9 locus from Pan-Ancestry Analysis, B. Chromosome 16 locus from Pan-Ancestry Analysis, C. Chromosome 2 locus from Pan-Ancestry Analysis, D. Chromosome 6 locus from Pan-Ancestry Analysis, E. Chromosome 9 locus for European-Ancestry Analysis, F. Chromosome 6 locus from European-Ancestry Analysis. (TIF) [file pgen.1010623.s010.tif]

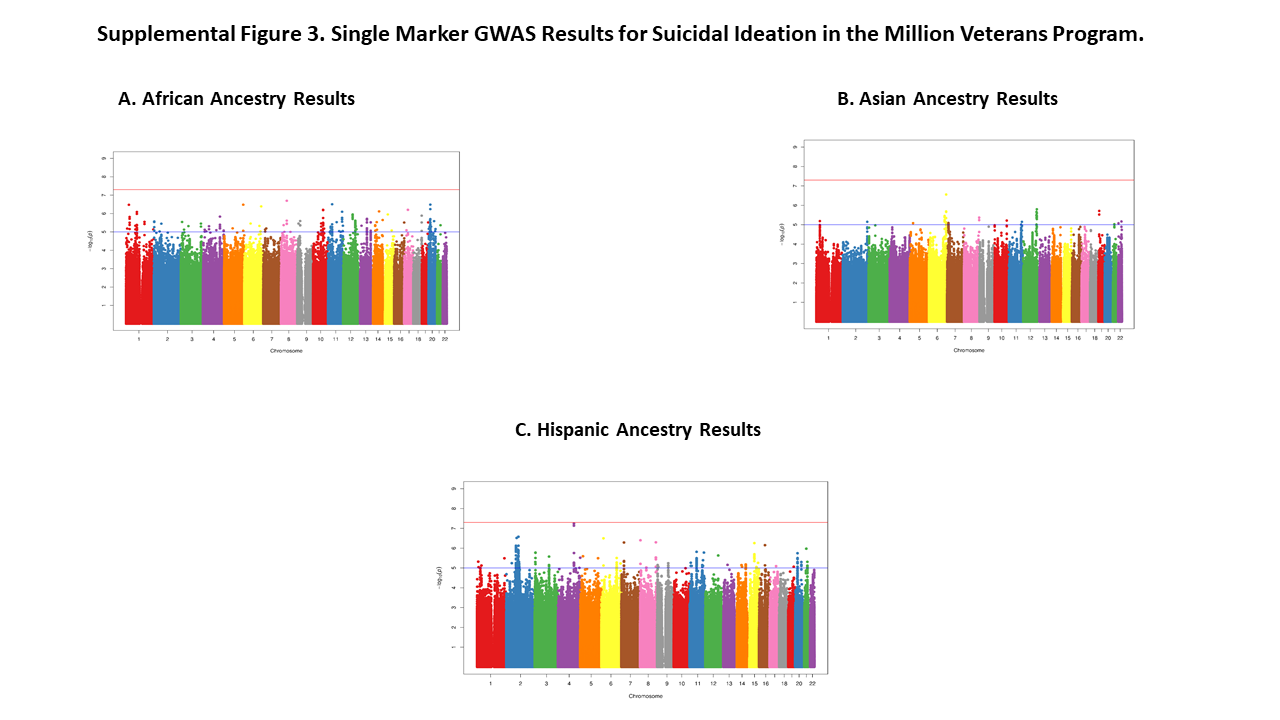

Supplement: S3 Fig — A. African Ancestry Results, B. Asian Ancestry Results, C. Hispanic Ancestry Results. (TIF) [file pgen.1010623.s011.tif]

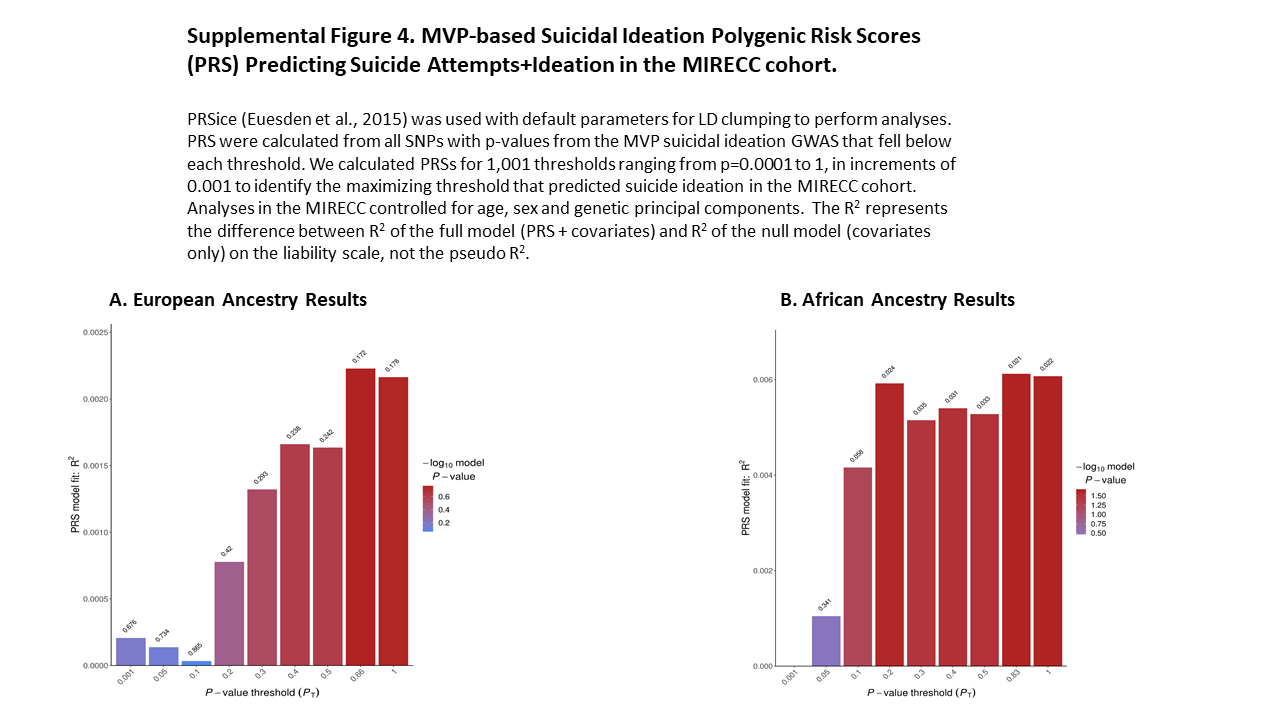

Supplement: S4 Fig — A. European Ancestry Results, B. African Ancestry Results. (TIF) [file pgen.1010623.s012.tif]
